# Morphological and molecular evidence reveal two new species of *Sutorius* (Boletaceae, Basidiomycota) from Guizhou Province, China

**DOI:** 10.3897/mycokeys.134.192749

**Published:** 2026-06-22

**Authors:** Xiu-Hong Xu, Xing Mo, A-Min Chen, Ya-Dong Li, Yun Pei, Jing-Wei Li, Ting-Chi Wen, Samantha C. Karunarathna, Entaj Tarafder, Wan-Ping Zhang

**Affiliations:** 1 College of Agriculture, Guizhou University, Guiyang 550025, China Center for Yunnan Plateau Biological Resources Protection and Utilization & Yunnan International Joint Laboratory of Fungal Sustainable Utilization in South and Southeast Asia, College of Biology and Food Engineering, Qujing Normal University Qujing China; 2 Institute of Vegetable Industry Technology Research, Guizhou University, Guiyang 550025, China College of Agriculture, Guizhou University Guiyang China; 3 Guiyang Vegetable Technology Extension Station, Guiyang 550081, China Institute of Vegetable Industry Technology Research, Guizhou University Guiyang China; 4 Guizhou Modern Seed Industry Group Co. Ltd., Guiyang 550000, China The Engineering Research Center of Southwest Bio-Pharmaceutical Resources, Ministry of Education, Guizhou University Guiyang China; 5 The Engineering Research Center of Southwest Bio-Pharmaceutical Resources, Ministry of Education, Guizhou University, Guiyang 550025, China Guiyang Vegetable Technology Extension Station Guiyang China; 6 Center for Yunnan Plateau Biological Resources Protection and Utilization & Yunnan International Joint Laboratory of Fungal Sustainable Utilization in South and Southeast Asia, College of Biology and Food Engineering, Qujing Normal University, Qujing 655099, China Guizhou Modern Seed Industry Group Co. Ltd. Guiyang China

**Keywords:** Boletales, ectomycorrhizal fungi, morphology, multigene phylogeny, taxonomy

## Abstract

*Sutorius* is a genus within the family Boletaceae, typically characterised by chocolate-brown to reddish-brown or slightly purplish colouration, finely scaly stems and reddish-brown spore deposits. Although numerous species of this genus have been reported from India and northern to north-eastern Thailand, its species diversity remains insufficiently explored. During an extensive survey on diversity of boletes in Zunyi City, Guizhou Province, China, this study identified two new *Sutorius* species, based on morphological and multi-locus (nrLSU, nrITS, *TEF1-α* and *RPB2*) phylogenetic analyses and they were assigned as *S. yuxiensis* and *S. rubropurpureus*. *Sutorius yuxiensis* is morphologically characterised by a yellowish-brown pileus with densely arranged pores, giving the surface an irregular appearance, along with a pale purplish-grey colouration. *Sutorius rubropurpureus* is morphologically characterised by a purplish-red to purplish-grey pileus, the pore surface is purplish-red to reddish-brown in colour and the presence of pleurocystidia. *S. yuxiensis* is phylogenetically close to *S. obscuripellis* and *S. ubonensis* and forms an independent lineage. *S. rubropurpureus* clusters with *S. rubinus* and related congeners. Detailed descriptions, illustrations, phylogenetic analyses and comparisons with closelyrelated taxa are provided to support the recognition of these two novel species.

## Introduction

*Sutorius* Halling, Nuhn, and N.A. Fechner belongs to the family Boletaceae and was established in 2012 to accommodate two bolete species, *Boletus eximius* Peck and *Leccinum australiense* Bougher and Thiers (Halling et al. 2012). Originally, Peck (1887) had described *Boletus eximius* Peck as a new name (Halling et al. 2012). Subsequently, Snell and Dick (1970), Smith and Thiers (1971) and Grund and Harrison (1976) also described this taxon, based on North American collections. Due to the absence of a clearly designated holotype, Halling (1983) later selected a specimen from the Frost Collection (North America) as the lectotype. Eventually, *Boletus eximius* was transferred to *Sutorius* and recognised as *Sutorius eximius* Peck (Bessette et al. 2016). Members of *Sutorius* are characterised by distinctive morphological features, including chocolate to reddish-brown or purplish–brown basidiocarps, a finely scaly stipe and a reddish-brown spore deposit, enabling its differentiation from other genera within *Pulveroboletus*Murrill (1909). With advances in molecular systematics, phylogenetic analyses, based on multiple gene regions *atp6*, *TEF1* and *RPB2*, have significantly advanced the taxonomic framework of *Sutorius*. This has not only facilitated the discovery of several new species, but also achieved breakthroughs in taxonomic revision. For instance, *Tylopilus maculatoides* Horak, previously classified within *Tylopilus* Karst, has been reclassified into *Sutorius*. This reclassification has further clarified the phylogenetic relationships amongst species within the genus (Karsten 1881; Horak 2011). Recent studies have reported several new species of *Sutorius* from Asia, America and other regions (Smith and Thiers 1971; Fulgenzi et al. 2007; Halling et al. 2012; Chai et al. 2019; Li and Yang 2021; Vadthanarat et al. 2021). To date, 29 species within this genus have been documented (https://www.indexfungorum.org/, accessed on 10 May 2026), with Southeast Asia accounting for the largest proportion, 17 species having been reported from China, establishing it as a core area of biodiversity hotspot (Vadthanarat et al. 2024).

Species of *Sutorius* (Boletaceae) are ectomycorrhizal fungi that play important ecological roles in forest ecosystems by forming mutualistic associations with a wide range of woody plants, particularly in tropical and subtropical regions (Smith and Read 2008; Tedersoo and Smith 2013). Through ectomycorrhizal symbiosis, Sutorius species enhance host plant nutrient uptake, especially nitrogen and phosphorus and improve resistance to environmental stress, while receiving photosynthetically derived carbon in return (Smith and Read 2008). Although the genus Sutorius has been primarily studied from a taxonomic and phylogenetic perspective (Vadthanarat et al. 2021), its ecological functions are considered comparable to other ectomycorrhizal members of Boletaceae, which are known to influence plant community structure and ecosystem stability (Tedersoo and Smith 2013).

Although the type species of *Sutorius eximius*, has been recorded in China and its taxonomic status confirmed through morphological and molecular evidence, dedicated surveys and systematic studies of this genus in Guizhou Province remain limited. In this study, boletoid fungi were collected from various locations in Guizhou Province. By integrating detailed morphological observations with phylogenetic analyses, based on nrITS, nrLSU, *TEF1–α* and *RPB2* gene regions, two new species within the genus *Sutorius* were discovered and described. The findings aim to enrich the species diversity data for *Sutorius* in China, laying the foundation for the precise conservation and sustainable utilization of fungal resources in Guizhou Province.

## Materials and methods

### Sample collection and morphological study

Specimens were collected from Yuxi Town, Daozhen County, Guizhou Province, China. The area has a humid subtropical monsoon climate, with vegetation consisting primarily of subtropical evergreen broadleaf forests, coniferous forests and secondary shrublands. Macroscopic morphological characteristics were documented using in situ photographs and detailed field notes were taken at the time of collection. Colour descriptions followed the standardised colour codes of Kornerup and Wanscher (1978). After recording the general macroscopic features, each specimen was cut longitudinally to observe and document any discoloration in the damaged tissue. Subsequently, the samples were dried in an oven at 45 °C for 10 h, sealed in a self-sealing bag containing desiccant and temporarily stored at 4 °C in a resource preservation facility. For microscopic examination, dried specimens were rehydrated, sectioned and mounted in a solution containing 5% potassium hydroxide (KOH) and 1% Congo red. Tissue samples were taken from the pileus along the radial direction (perpendicular to the surface) at the mid-point between the centre and margin, while stipe tissues were sampled from the mid-point along the longitudinal axis (Li et al. 2011; Hosen et al. 2013; Zhu et al. 2015). Microscopic structures, including basidia, basidiospores and cystidia, were examined and photographed using a Nikon Eclipse Ni compound microscope, equipped with a Nikon Digital Sight 10 imaging system. Measurements were obtained from 30–50 randomly selected basidiospores and 20 additional microscopic structures. Basidiospore dimensions are presented as (a) b–c (d), where b–c represents the 95% confidence interval of the measured range and “a” and “d” denote the extreme values. The symbol Q refers to the length–to–width ratio of an individual basidiospore, while Qm represents the mean Q value ± standard deviation. All examined specimens were deposited in the Herbarium of Cryptogams at the Kunming Institute of Botany, Chinese Academy of Sciences (**HKAS**).

### DNA extraction, Polymerase Chain Reaction (PCR) amplification and sequencing

The DNA of the dry specimens was extracted using the Ezup Column Fungal Genomic DNA Extraction Kit. PCR was performed using the nuclear ribosomal large subunit (nrLSU), the internal transcribed spacer (ITS) region, the translation elongation factor 1–α (*TEF1–α*) and the second largest subunit of RNA polymerase II (*RPB2*). The nrLSU region was amplified using the primers LR0R–F and LR5–R (Vilgalys and Hester 1990). The nrITS region was amplified with primers ITS1 and ITS4 (White et al. 1990), *TEF1–α* with primers EF1–2F and EF1–2R (Rehner and Buckley 2005) and *RPB2* with primers bRPB2–6F and bRPB2–7.1R (Matheny 2005). All primers were synthesised by Shanghai Sangon Biotech Co., Ltd. Polymerase chain reaction (PCR) amplifications were carried out in a total volume of 20 μl, consisting of 10 μl of 2× Taq PCR StarMix, 1 μl of each primer, 1 μl of DNA template and 7 μl of double–distilled water (ddH_2_O). The PCR products were examined by electrophoresis on 1% (w/v) agarose gels. Successfully amplified products were excised and purified using the Agarose Gel DNA Recovery Kit (CWBIO) in accordance with the manufacturer’s instructions. Purified PCR products were subsequently sequenced by Shanghai Sangon Biotech Co., Ltd.

### Sequence alignment and phylogenetic analyses

After quality assessment, sequences with obvious background peaks or contamination were excluded. Forward and reverse sequences were assembled using SEQMAN software and the assembled sequences were then submitted to GenBank for BLAST homology searches to obtain closely-related reference sequences (Table 1). Subsequently, the sequences generated in this study and the retrieved reference sequences were aligned using the online version of MAFFT 7.0 (http://mafft.cbrc.jp/alignment/server/) (Katoh and Standley 2013), with manual adjustments made in BIOEDIT v.7.0.9 (Hall 1999) as needed. SequenceMatrix (Vaidya et al. 2011) was used to assemble the sequences of the four genes. The Maximum Likelihood phylogenetic tree was constructed using RAxML–HPC2 on ACCESS (8.2.12) of the CIPRES Science Gateway platform, applying the GTRCAT nucleotide substitution model with 1,000 bootstrap replicates (Miller et al. 2010). For Bayesian Inference (BI), MrBayes version ACCESS (3.2.7a) was selected for execution on the CIPRES portal. Optimal model selection for each data partition was performed using MrModelTest v. 2.0.1 (Nylander 2004), estimating the optimal phylogenetic model for each subset. Phylogenetic trees were constructed using the Akaike Information Criterion (AIC), ultimately selecting the GTR+I+G model for all gene regions. Three Markov chains were run, each undergoing 5 million generations of evolution, with sampling every 100 generations until the mean standard deviation reached 0.01. After discarding the top 20% of the evolved trees, the remaining trees were aggregated to calculate posterior probabilities (PP) for each branch. The final phylogenetic tree was visualised and optimised using FigTree v.1.4.0 software (Rambaut et al. 2014).

**Table 1.** List of collections used for DNA analyses, with origin, GenBank accession numbers and reference(s).

Species	Voucher	Origin	GenBank accession numbers				Reference(s)
			nrLSU	nrITS	*TEF1–α*	RPB2	
***Boletus edulis***	**HMJAU4637**	**Russia**	**KF112455**	**–**	**KF112202**	**KF112704**	**Wu et al. (2014)**
***Boletus monilifer***	**HKAS83098**	**China**	**KM820807**	**–**	**–**	**–**	**Cui et al. (2016)**
***Boletus monilifer***	**HKAS83205**	**China**	**KM820806**	**–**	**–**	**–**	**Cui et al. (2016)**
***Boletus reticuloceps***	**HKAS51232**	**China**	**KT990537**	**–**	**KT990739**	**KT990376**	**Wu et al. (2016)**
***Boletus reticuloceps***	**HKAS57671**	**China**	**KF112454**	**–**	**KF112201**	**KF112703**	**Wu et al. (2014)**
***Caloboletus firmus***	**MB06–060**	**USA**	**–**	**–**	**KF030408**	**–**	**Nuhn et al. (2013)**
***Caloboletus firmus***	**NY00796115**	**China**	**KJ605678**	**KJ605656**	**KJ619464**	**–**	**Zhao et al. (2014)**
***Caloboletus guanyui***	**FHMU_2019**	**China**	**MH879708**	**MH885365**	**MH879734**	**MH879751**	**Chai et al. (2019)**
***Caloboletus guanyui***	**FHMU_2040**	**China**	**MH879709**	**MH885366**	**MH879736**	**MH879752**	**Chai et al. (2019)**
***Caloboletus yunnanensis***	**HKAS69214**	**China**	**KJ184556**	**KJ605663**	**KJ184568**	**–**	**Zhao et al. (2014)**
***Caloboletus yunnanensis***	**HKAS58694**	**China**	**KJ605672**	**KJ605664**	**KJ619470**	**–**	**Zhao et al. (2014)**
***Neoboletus sanguineoides***	**HKAS74733**	**China**	**KT990606**	**–**	**KT990800**	**KT990441**	**Wu et al. (2016)**
***Neoboletus sanguineoides***	**HKAS63530**	**China**	**KT990607**	**–**	**KT990801**	**–**	**Wu et al. (2016)**
***Neoboletus sanguineus***	**HKAS80823**	**China**	**KT990608**	**–**	**KT990802**	**KT990442**	**Wu et al. (2016)**
***Neoboletus sanguineus***	**HKAS90211**	**China**	**KT990610**	**–**	**KT990804**	**KT990444**	**Wu et al. (2016)**
***Neoboletus tomentulosus***	**HKAS77614**	**China**	**–**	**–**	**KT990805**	**KT990445**	**Wu et al. (2016)**
***Neoboletus venenatus***	**HKAS63535**	**China**	**–**	**–**	**KT990807**	**KT990448**	**Wu et al. (2016)**
***Rubroboletus rhodosanguineus***	**4252**	**USA**	**KF030252**	**–**	**KF030412**	**–**	**Nuhn et al. (2013)**
***Rubroboletus sinicus***	**HKAS 68620**	**China**	**KF112319**	**KJ951991**	**KF112146**	**KF112661**	**Wu et al. (2014)**
***Rubroboletus sinicus***	**HKAS56304**	**China**	**KJ605673**	**KJ605666**	**KJ619483**	**–**	**Zhao et al. (2014)**
***Sutorius aff. eximius***	**HKAS 56291**	**China**	**KF112400**	**–**	**KF112208**	**KF112803**	**Wu et al. (2014)**
***Sutorius aff. eximius***	**S.D.Yang010**	**China**	**MH879697**	**MH885359**	**MH879727**	**–**	**Chai et al. (2019)**
***Sutorius apleurocystidiatus***	**KD 23–017**	**India**	**–**	**–**	**PP855520**	**PP855518**	**Das et al. (2024)**
***Sutorius apleurocystidiatus***	**KD 23–015**	**India**	**–**	**–**	**PP855519**	**PP855517**	**Das et al. (2024)**
***Sutorius alpinus***	**HKAS50420**	**China**	**KT990549**	**–**	**KT990750**	**KT990387**	**Wu et al. (2016)**
***Sutorius alpinus***	**HKAS59657**	**China**	**KT990707**	**–**	**KT990887**	**KT990505**	**Wu et al. (2016)**
***Sutorius alpinus***	**HKAS 52672**	**China**	**KF112399**	**–**	**KF112207**	**KF112802**	**Wu et al. (2014)**
***Sutorius australiensis***	**REH9280**	**Australia**	**JQ327005**	**–**	**JQ327031**	**–**	**Halling et al. (2012)**
***Sutorius australiensis***	**REH9441**	**Australia**	**JQ327006**	**–**	**JQ327032**	**MG212652**	**Halling et al. (2012)**
***Sutorius eximius***	**TWO986**	**Costa Rica**	**JQ327009**	**–**	**JQ327028**	**–**	**Halling et al. (2012)**
***Sutorius eximius***	**JLF2547**	**Unites States**	**KC812314**	**KC812313**	**–**	**–**	**Halling et al. (2012)**
***Sutorius eximius***	**NY:02449711**	**Unites States**	**MK601813**	**–**	**MK721167**	**MK766369**	**Kuo and Ortiz-Santana (2020)**
***Sutorius eximius***	**REH9400**	**Unites States**	**JQ327004**	**–**	**JQ327029**	**MG212653**	**Halling et al. (2012)**
***Sutorius eximius***	**REH8594**	**Costa Rica**	**JQ327008**	**–**	**JQ327027**	**–**	**Halling et al. (2012)**
***Sutorius eximius***	**TWO995**	**Costa Rica**	**JQ327010**	**–**	**JQ327030**	**–**	**Halling et al. (2012)**
***Sutorius eximius***	**NY:1393562**	**Costa Rica**	**–**	**–**	**MK721180**	**MK766383**	**Kuo and Ortiz-Santana (2020)**
***Sutorius eximius***	**MyCoPortal 03817572**	**United States**	**–**	**MW899061**	**–**	**–**	**Halling et al. (2012)**
***Sutorius eximius***	**HKAS91261**	**China**	**–**	**–**	**–**	**MT110444**	**Li and Yang (2021)**
***Sutorius maculatoides***	**OR0758**	**Thailand**	**–**	**–**	**MN067481**	**MN067498**	**Vadthanarat et al. (2021)**
***Sutorius maculatoides***	**OR0626**	**Thailand**	**–**	**–**	**MN067480**	**–**	**Vadthanarat et al. (2021)**
***Sutorius microsporus***	**HKAS 68720**	**China**	**NG_088137**	**–**	**–**	**–**	**Li and Yang (2021)**
***Sutorius mucosus***	**OR0851**	**Thailand**	**–**	**–**	**MN067483**	**MN067499**	**Vadthanarat et al. (2021)**
***Sutorius obscuripellis***	**Wu2070**	**China**	**MT154772**	**–**	**MW165273**	**–**	**Vadthanarat et al. (2021)**
***Sutorius obscuripellis***	**OR0949**	**Thailand**	**–**	**–**	**MN067494**	**MN067510**	**Vadthanarat et al. (2021)**
***Sutorius pachypus***	**SV0098**	**Thailand**	**–**	**–**	**MN067485**	**MN067501**	**Vadthanarat et al. (2021)**
***Sutorius pachypus***	**OR0411**	**Thailand**	**–**	**–**	**MN067484**	**MN067500**	**Vadthanarat et al. (2021)**
***Sutorius pseudotylopilus***	**Wu499**	**China**	**MT154774**	**–**	**–**	**–**	**Li and Yang (2021)**
***Sutorius pseudotylopilus***	**Wu938**	**China**	**MT154775**	**–**	**–**	**–**	**Li and Yang (2021)**
***Sutorius pseudotylopilus***	**SV0401**	**Thailand**	**–**	**–**	**MN067486**	**MN067502**	**Vadthanarat et al. (2021)**
***Sutorius pseudotylopilus***	**SV0415**	**Thailand**	**–**	**–**	**MN067487**	**MN067503**	**Vadthanarat et al. (2021)**
***Sutorius pseudotylopilus***	**OR0378B**	**Thailand**	**–**	**–**	**MH614740**	**MH614787**	**Vadthanarat et al. (2021)**
***Sutorius rubropurpureus***	**HKAS151559**	**China**	**PX630357**	**PX630361**	**PX646528**	**PX646532**	**This study**
***Sutorius rubropurpureus***	**HKAS151560**	**China**	**PX630358**	**PX630362**	**PX646529**	**PX646533**	**This study**
***Sutorius rubinus***	**OR0403**	**Thailand**	**–**	**–**	**MN067488**	**MN067504**	**Vadthanarat et al. (2021)**
***Sutorius rubinus***	**OR0409**	**Thailand**	**–**	**–**	**MN067489**	**MN067505**	**Vadthanarat et al. (2021)**
***Sutorius subrufus***	**N.K.Zeng3043**	**China**	**MH879698**	**MH885360**	**MH879728**	**MH879745**	**Chai et al. (2019)**
***Sutorius subrufus***	**N.K.Zeng3045**	**China**	**MH879699**	**MH885361**	**MH879729**	**MH879746**	**Chai et al. (2019)**
***Sutorius ubonensis***	**SV0203**	**Thailand**	**–**	**–**	**MN067492**	**MN067508**	**Vadthanarat et al. (2021)**
***Sutorius ubonensis***	**SV0032**	**Thailand**	**–**	**–**	**MN067491**	**MN067507**	**Vadthanarat et al. (2021)**
***Sutorius vellingae***	**ECV3603**	**Thailand**	**JQ327000**	**–**	**JQ327033**	**–**	**Halling et al. (2012)**
***Sutorius yuxiensis***	**HKAS151561**	**China**	**PX630359**	**–**	**PX646530**	**PX646534**	**This study**
***Sutorius yuxiensis***	**HKAS151562**	**China**	**PX630360**	**–**	**PX646531**	**PX646535**	**This study**

Note: ‘–‘ refers to the missing data.

## Results

### Phylogenetic analyses

**Figure 1.**
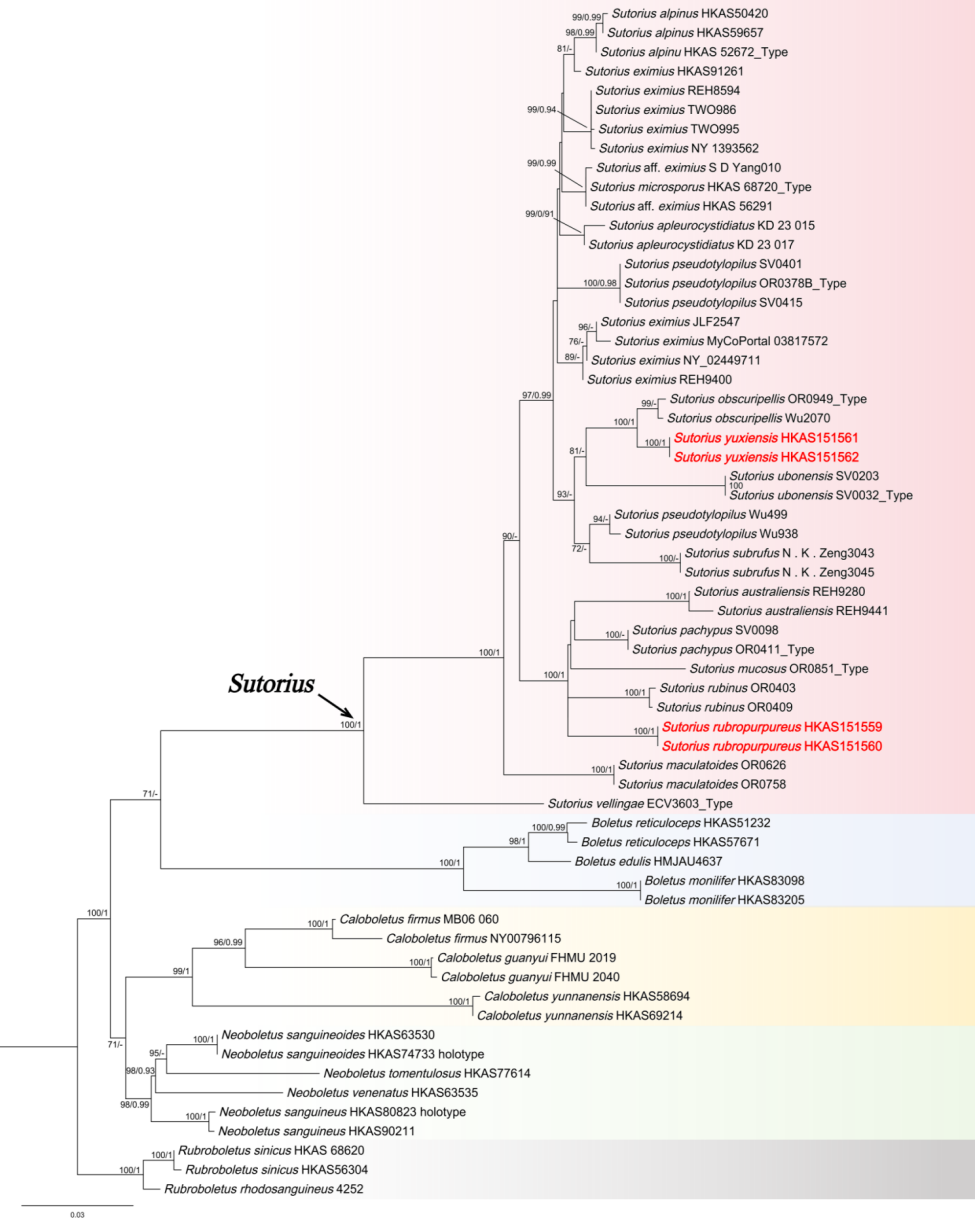
The phylogram was constructed using Maximum Likelihood and Bayesian Inference methods, based on an integrated dataset (nrLSU, nrITS *TEF1–α* and *RPB2*). The figure depicts the ML tree. Bootstrap support values (BS ≥ 70%) and posterior probabilities (PP ≥ 0.90) are shown above the supported branches. New species are placed in bold red font to highlight their phylogenetic positions in the tree.

Fourteen new sequences were obtained and deposited in GenBank (Table 1). The alignment matrix based on four molecular markers (nrLSU, nrITS, *TEF1–α* and RPB2) spanned 2,869 base pairs (bp) (including 839 bp for nrLSU, 695 bp for nrITS, 608 bp for *TEF1–α*, and 727 bp for *RPB2* sites) with gaps. The study employed *Rubroboletus sinicus* Chiu and *Rubroboletus rhodosanguineus* Both (Zhao and Wu 2014) as the outgroup. Phylogenetic trees constructed using Maximum Likelihood (ML) and Bayesian Inference (BI) methods showed only minor differences in statistical support values and were highly consistent. Therefore, this paper presents only the ML tree (Fig. 1).

The phylogenetic tree constructed from the dataset (Fig. 1) shows that *Sutorius yuxiensis* and *S. obscuripellis* Vadthanarat are sister taxa (including its type sequences OR0949 and Wu2070) and this branch is strongly supported (MLBS = 100%, PP = 1.00). This new species is closely related to *S. obscuripellis* and *S. ubonensis* Vadthanarat, but it represents an independent evolutionary lineage. The clade containing *S. rubropurpureus*, together with the branches of *S. rubinus* Vadthanarat (OR0403 and OR0409), collectively form a major clade. This major clade is placed alongside the branches of species, such as *S. mucosus* Vadthanarat, *S. pachypus* Vadthanarat and *S. australiensis* Bougher & Thiers (MLBS = 100%, PP = 1.00), constituting an independent evolutionary lineage within *Sutorius*. However, the low support for the branch containing *S. rubinus* indicates that its phylogenetic relationship with *S. rubinus* is not strongly supported in the current analysis. Molecular evidence indicates that it represents a new species within *Sutorius*, closely related to the aforementioned species, but clearly divergent.

### Taxonomy

#### Sutorius yuxiensis

Taxon classificationFungiBoletalesBoletaceae

X.H. Xu, X. Mo & W.P. Zhang sp. nov.

5FC67EFE-AC0F-5307-810E-84636788C5DD

861515

Fig. 2

##### Etymology.

The specific epithet ‘yuxiensis’ after to the type locality, Yuxi Town.

**Figure 2.**
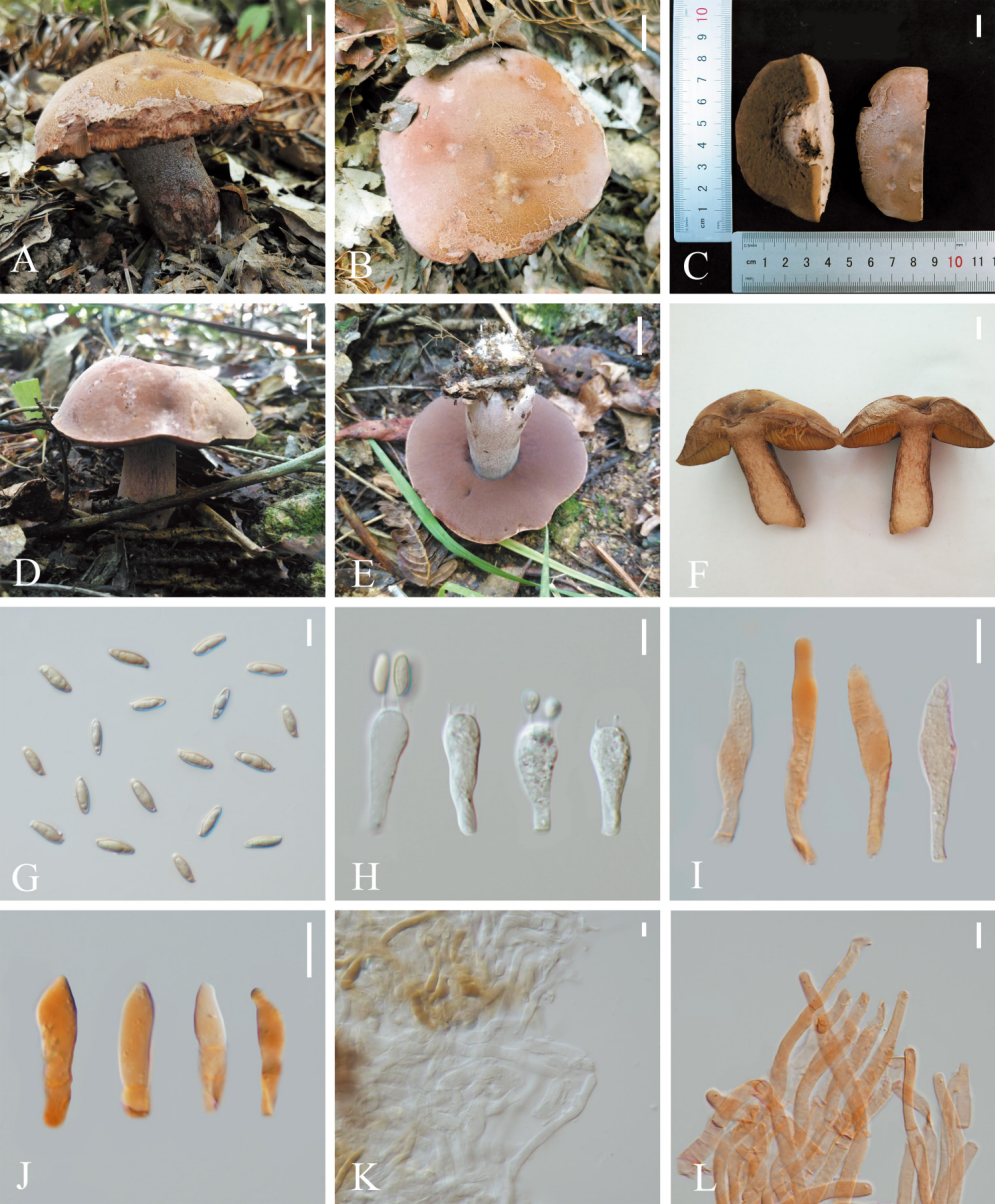
**A–F**. Basidiomata of *S. yuxiensis* in nature and indoor cross–section diagrams. (**A–C**. HKAS151561; **D–F**. HKAS151562); **G**. Basidiospores; **H**. Basidia; **I**. Pleurocystidia; **J**. Cheilocystidia; **K**. Pileipellis; **L**. Stipitipellis. Scale bars: 10 mm (**A–F**); 10 µm (**G–L**). All micrographs were taken from the holotype.

##### Holotype.

China • Guizhou Province, Zunyi City, Daozhen County, Yuxi Town, 28°50'42"N, 107°37'32"E, 1184.74 m alt., 16 August 2019, Xiu-hong Xu (holotype: HKAS151561).

##### Diagnosis.

Compared to its sister species *S. obscuripellis*, the new species exhibits smaller cheilocystidia and has pleurocystidia; the pileipellis is composed of brown to dark brown hyphae, colourless to brownish-yellow to pale brown in KOH, whereas *S. obscuripellis* has the pileipellis composed of brown to pale yellow hyphae in water, exhibiting slight extracellular brown pigmentation; less brown in KOH.

##### Description.

***Basidiomata*** medium sized, Pileus 40–65 mm in diam., convex to slightly convex, with slightly deflexed, becoming straight margin; surface smooth, densely covered with fine, dense hairs, yellowish-brown (7D6), with patchy colour variation, irregularly distributed spots, the margin sometimes fading in colour. ***Context*** 5–10 mm thick halfway to the margin, yellowish-white (4A2), with scattered small groups of dark reddish-brown (9F5–6) encrustations, unchanging or slightly reddening when cut. ***Stipe*** 40–60 × 15–20 mm; clavate to cylindrical, surface dull, even to finely scabrous, violet-grey (18D2) with violet-brown (10E3–4) granulose squamules; basal tomentum off-white to yellowish-white. Stipe context solid, fleshy, fibrous, yellowish-white (5C6), with a dark brown hue near the edges (6E5–6), with scattered small groups of reddish-brown (9E4–5) encrustations. ***Hymenophore*** tubulose, 5–10 mm long, adnexed to adnate, depressed around stipe, ventricose; tubes orange-grey (6B2), easily separable. ***Pores*** 0.2–0.5 mm wide at mid-radius, roundish, closely packed, with subregular pore surface, pale violet-grey (9–10B3). Odour, taste and spore print not recorded.

***Basidiospores*** (10.33–)10.49–14.90(–14.95) × (3.13–)3.26–5.40(–5.43) μm [Q = (2.23–)2.30–3.99(–4.40) μm, Qm = 2.93 ± 0.41 μm, n = 30], narrowly ellipsoid to subcylindrical, thin-walled, smooth, brownish to yellowish hyaline in water, yellowish hyaline in KOH, inamyloid. ***Basidia*** (21.04–)21.31–34.00(–34.05) × (6.83–)6.90–10.65(–10.66) µm, 4-spored, with sterigmata 3–5 × 0.5–0.8 µm, clavate, hyaline and inamyloid. ***Cheilocystidia*** (14.69–)14.83–25.56(–25.64) × (4.08–)4.10–6.76(–6.99) µm, narrowly fusiform to fusiform, smooth, thin–walled. ***Pleurocystidia*** (36.56–)37.00–48.50(–48.75) × (5.84–)6.00–9.30(–9.47) µm, narrowly fusiform, thin–walled, hyaline. Trama divergent, hyphae thin-walled, septate. ***Pileipellis*** an intricate trichoderm, 100–160 µm thick, composed of loosely interwoven apical cells 18–65 × 3–6 µm, with asubacute to rounded apex, pale yellow to hyaline in water, exhibiting slight extracellular brown pigmentation; less brown in KOH. ***Stipitipellis*** 60–100 µm thick, composed of parallel hyphae, composed of thin-walled hyaline hyphae; terminal cells of hyphae 16–30 × 6–8 µm, cystidioid, broadly clavate or ventricose. All hyphae show no chromogenic reaction in Melzer’s. Clamp connections not seen in any tissue.

#### Sutorius rubropurpureus

Taxon classificationFungiBoletalesBoletaceae

X.H. Xu, X. Mo & W.P. Zhang sp. nov.

E0126A5F-1B9A-554D-A727-C056FC84FB11

861513

Fig. 3

##### Etymology.

The specific epithet rubropurpureus>’ is a compound adjective derived from the Latin roots rubro- (meaning “red”) and purpureus (meaning “purple”), referring to the distinctive reddish–purple colouration of the basidiocarps, particularly the pileus and stipe surfaces.

**Figure 3.**
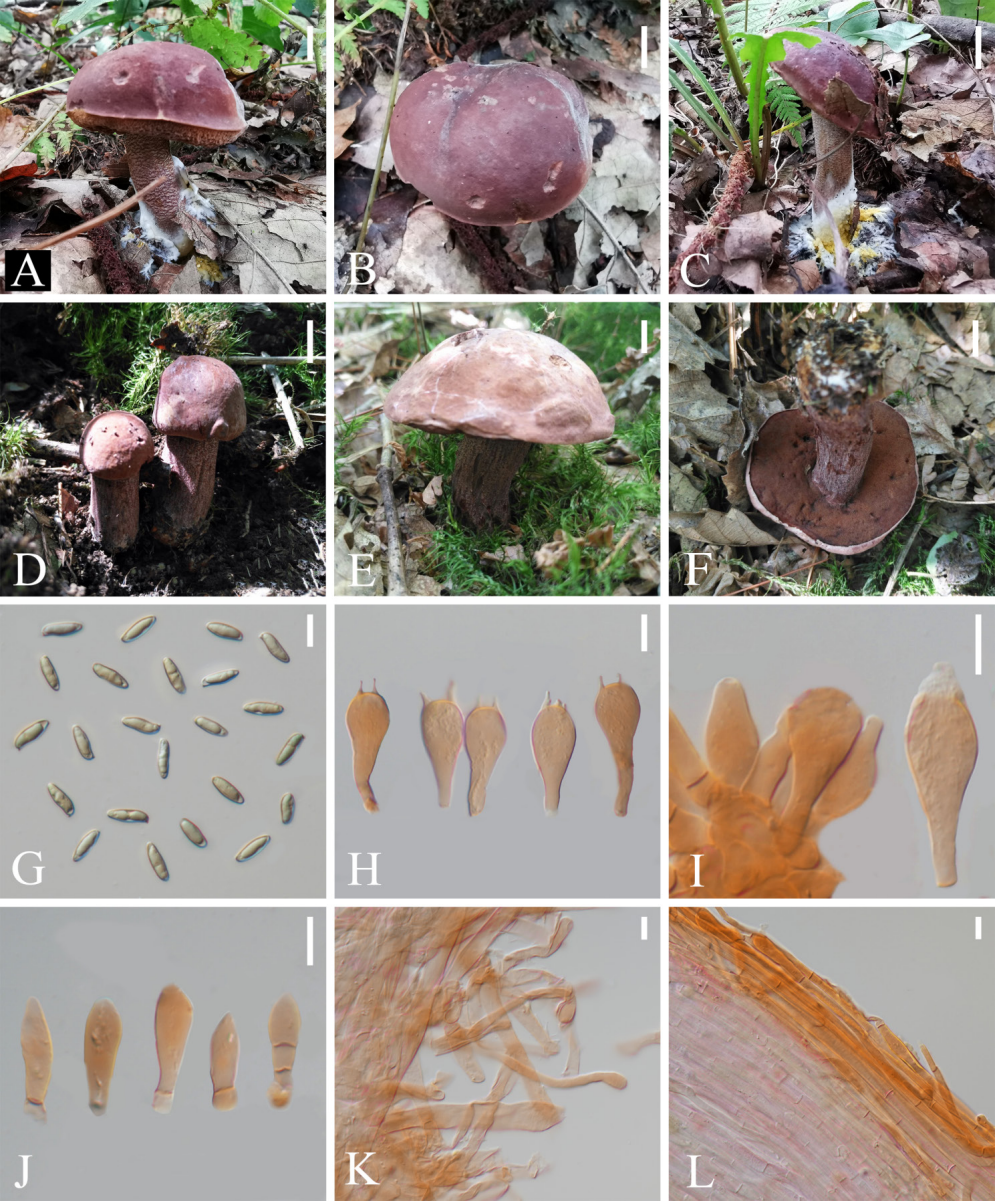
**A–F**. Basidiomata of *S. rubropurpureus* in nature. (**A–C**. HKAS151559; **D–F**. HKAS151560); **G**. Basidiospores; **H**. Basidia; **I**. Pleurocystidia; **J**. Cheilocystidia; **K**. Pileipellis; **L**. Stipitipellis. Scale bars: 10 mm (**A–F**); 10 µm (**G–L**). All micrographs were taken from the holotype.

##### Holotype.

China • Yuxi Town, Daozhen County, Zunyi City, Guizhou Province, 28°50'41"N, 107°37'34"E, 1176.29 m alt., 21 September 2019, A-min Chen and Ya-dong Li (holotype: HKAS151559).

##### Diagnosis.

The newly-described taxa is characterised by medium-sized basidiomata, purplish-red to purplish-grey; with pleurocystidia; pore surface is purplish-red to reddish-brown, with circular pores. They turn slightly yellow when wetted and transparent to pale yellow in KOH. These characteristics distinguish it from closely-related species *S. rubinus*, *S. mucosus*, *S. pachypus* and *S. australiensis*.

##### Description.

***Basidiomata*** medium–sized. ***Pileus*** 40–80 mm in diam., hemispherical, with slightly deflexed, becoming straight margin, surface at first subrugulose, tomentose, the original purplish-red (13E5–6) has aged to a slightly purplish–grey to brown (13C4, 7E5), gradually paler to the margin. ***Context*** 4–10 mm thick halfway to the margin, pale-yellow (4A2). ***Stipe*** is 30–50 × 10–20 mm, terete, cylindrical to subclavate or tapering downwards, sometimes with sub-bulbous base, reddish-brown (10D5–10E5), injured without changing colour; surface finely scabrous, densely covered with greyish-white (10B1–2) granulose squamules; basal tomentum white to off-white (14B1–2). ***Hymenophore*** tubulose, adnexed to narrowly adnexed, ventricose, slightly depressed around the stipe. The surface of the tubes appears dark purple (12E4); tubes orange-brown (7E7), 3–10 mm long halfway to the margin. Odour and taste not recorded. Spore print not recorded.

***Basidiospores*** (9.85–)9.90–15.20(–15.48) × (3.57–)3.60–5.50(–5.86) μm [Q = (2.27–)2.30–3.49(–3.55) μm, Qm = 2.86 ± 0.37 μm, n = 30], subfusoid, inequilateral in side view, smooth, yellowish hyaline in KOH, inamyloid. ***Basidia*** (25.28–)25.50–32.00(–32.37) × (8.07–)8.20–12.80(–12.99) μm, clavate, 4-spored; sterigmata 2–4 × 0.8–1.5 µm. ***Cheilocystidia*** (14.60–)14.80–24.50(–24.83) × (4.27–)4.49–6.60(–6.79) µm, frequent, narrowly fusiform to fusiform with subacute apex, thin-walled. ***Pleurocystidia*** (20.23–)20.50–33.89(–34.08) × (4.43–)4.60–11.45(–11.77) µm, spindle-shaped, smooth, thin-walled. ***Pileipellis*** an intricate trichoderm to a slightly gelatinised intricate trichoderm, made of densely interwoven, thin-walled, smooth, 100–130 µm thick, hyaline hyphae 3–10 µm wide. Terminal cells of hyphae 19–30 × 4–11 µm, cylindrical to subcylindrical, subventricose to subfusiform, rarely inflated to clavate. They turn slightly yellow when wetted and become transparent to pale yellow when treated with KOH. ***Stipitipellis*** 30–60 µm thick, with a thin layer of gluten, hymeniform, composed of thin-walled, erect hyphae, with brown extracellular pigment; terminal cells of hyphae 17–40 × 4–8 µm, cystidioid, broadly clavate or ventricose. All hyphae show no chromogenic reaction in Melzer’s. Clamp connections absent in all tissues.

## Discussion

The taxonomic instability within *Sutorius* since its establishment in 2012 highlights the complementary yet complex roles of morphological characteristics and molecular evidence in delimiting taxa within the Boletaceae (Table 2). The type species, *S. eximius*, exhibits stipe characteristics that are superficially similar to those of *Leccinum*Gray (1821); however, these similarities lack developmental homology with members of that genus. Moreover, *S. eximius* does not represent a single, widely distributed or geographically isolated species, but, instead, constitutes a complex of closely-related taxa. Morphologically similar members of this complex have been demonstrated to represent distinct species originating from different continents or regions (Halling et al. 2012; Vadthanarat et al. 2021; Das et al. 2024). For *Tylopilus balloui*, molecular evidence supports its placement within *Tylopilus*, despite its basidiospore morphology deviating from the diagnostic characteristics traditionally associated with this genus (Osmundson and Halling 2010). Similarly, certain Asian collections exhibit morphological traits resembling those of *Sutorius* species, but consistently cluster within the *Tylopilus* lineage in molecular phylogenetic analyses. These discrepancies suggest convergent evolution or cryptic speciation and highlight the need to resolve such “morphological-molecular conflicts” through integrative analyses of multiple genetic loci (Chai et al. 2019; Vadthanarat et al. 2021). Comparable challenges have been reported between *Sutorius* and *Neoboletus* Gelardi, Simonini and Vizzini (2014), whose species often exhibit substantial overlap in basidiomata colouration and basidiospore ornamentation, rendering morphology–based identification unreliable. In contrast, multilocus phylogenetic analyses, particularly those incorporating *TEF1–α* and *RPB2* gene regions, have proven effective in resolving these conflicts (Chai et al. 2019). Notably, the *RPB2* fragment used in the present study has been shown to provide exceptionally high discriminatory power at both the genus and species levels within the Boletaceae, substantially outperforming the ITS region alone and thereby strengthening the robustness of taxonomic inferences (Wu et al. 2016).

**Table 2.** Major morphological comparison between the newly-described *Sutorius* species in this study and its closely-related taxa.

Species	Pileus length (mm)	Pileus Colour	Stipe (mm)	Stipe colour	Cystidia (µm)	Pileipellis (µm)	Reference
***S. yuxiensis***	40–65	Yellowish-brown (7D6)	40–60 × 15–20	Violet-grey (18D2) with violet-brown (10E3–4) granulose squamules	Cheilocystidia (14.69)14.83–25.56(25.64) × (4.08)4.10–6.76(6.99), Pleurocystidia (36.56)37.00–48.50(48.75) × (5.84)6.00–9.30(9.47)	100–160, thick, pale yellow to hyaline in water, less brown in KOH	**This study**
***S. obscuripellis***	(35) 45–60	Violet-brown to dark brown (10F5–6 to 9E4)	(35) 38–55 × 8–17	violet-grey (18D2, 16E2) with violet-brown (10E3–5) granulose squamules	Cheilocystidia (18) 19–24–30 (31) × (6) 6–7–9 (–9), Pleurocystidia absent	100–170, thick, brown to dark in water, less brown in KOH	**Vadthanarat et al. (2021)**
***S. rubropurpureus***	40–80	Purplish-red (13E5–6), slightly purplish-grey to brown (13C4, 7E5)	30–50 × 10–20	Reddish-brown (10D5 to 10E5), with greyish-white (10B1–2) granulose squamules	Cheilocystidia (14.60)14.80–24.50(24.83) × (4.27)4.49–6.60(6.79), Pleurocystidia (20.23)20.50–33.89(34.08) × (4.43)4.60–11.45(11.77)	100–130, thick, hyaline in water, pale yellow in KOH	**This study**
***S. rubinus***	45–75	reddish dark brown (9F4–6), reddish dark brown (8F5–8) to reddish-brown (8D/E6–8)	50–65 × 8–22	Reddish-grey to greyish brown (8 D/E 2–3), reddish-brown to dark brown (8F4–5) granulose squamules	Cheilocystidia (20) 20–26–32 (32) × (5) 5–7–8 (–8), Pleurocystidia absent	60–110, thick, slightly yellowish to reddish pale brown in water, mostly hyaline to yellowish pale brown in KOH	**Vadthanarat et al. (2021)**

From a taxonomic perspective, *S. yuxiensis* and *S. rubropurpureus* align with the shared characteristics of *Sutorius*. *Sutorius yuxiensis* exhibits chocolate-coloured basidiomata, greyish-brown hymenium, and flesh displaying visible mottled reddish-brown crusting, with narrowly elliptical basidiospores. *Sutorius rubropurpureus* exhibits reddish-brown hymenium and a stem surface with transversely fissured scales. Consequently, both *S. yuxiensis* and *S. rubropurpureus* are identified as belonging to the *Sutorius* through morphological and molecular characterisation. However, there are differences between species. Compared to *S. yuxiensis*, its sister clade *S. obscuripellis* exhibits the following morphological distinctions: *S. obscuripellis* possesses a darker brown cap, lacks lateral cystidia and its stem cross-section lacks a dark brown margin. Phylogenetic analysis indicates that *S. rubropurpureus* forms a major clade with *S. rubinus*, *S. mucosus*, *S. pachypus* and *S. australiensis*. However, these species differ morphologically from *S. rubropurpureus*: the basidiocarps of *S. rubinus* are dark reddish-brown to reddish-brown, in KOH, dark brown to black on the stipe and reddish on the hymenophore, lacking Pleurocystidia; *S. mucosus* has a waxy to subviscid pileus with an ixotrichoderm forming the pileipellis, composed of gelatinised hyphae and the lack of cheilocystidia and pleurocystidia; *S. pachypus* typically possesses a broader stipe (2.5–4 cm in width), with granulose squamules (Vadthanarat et al. 2021); *S. australiensis* has dark violet-brown pores when young, Stem has finely subsquamulose to finely scabrous–scissurate (Halling et al. 2012).

This study describes two new species of *Sutorius* from Zunyi City, Guizhou Province, China, based on an integrative approach combining morphological evidence with multilocus phylogenetic analyses of the ITS, nrLSU, *TEF1–α* and *RPB2* regions. The recognition of these new taxa further demonstrates that the diversity of Boletaceae in tropical and subtropical regions of Asia remains substantially underestimated, consistent with recent reports of concentrated discoveries of novel *Boletus* species across multiple regions of China (Das et al. 2023). Beyond expanding the known species diversity of *Sutorius* within subtropical karst forest ecosystems, these findings provide additional insights into the genus’s taxonomic delimitation, biogeographic patterns and evolutionary relationships.

## Supplementary Material

XML Treatment for Sutorius yuxiensis

XML Treatment for Sutorius rubropurpureus

## Additional information

### Artificial Intelligence (AI) use

The authors accept full responsibility for the content of the manuscript, including the disclosure of any use of AI.

No AI tools were used in the preparation of this manuscript.

### Author ORCIDs

X.-H. Xu https://orcid.org/0000-0002-6602-4246

X. Mo https://orcid.org/0009-0003-0295-5062

A.-M. Chen https://orcid.org/0000-0002-0412-2107

Y.-D. Li https://orcid.org/0009-0005-0470-0850

Y. Pei https://orcid.org/0000-0001-7730-4561

J.-W. Li https://orcid.org/0009-0002-4342-5837

T.-C. Wen https://orcid.org/0000-0003-1744-5869

S.C. Karunarathna https://orcid.org/0000-0001-7080-0781

E. Tarafder https://orcid.org/0000-0002-3680-3433

W.-P. Zhang https://orcid.org/0000-0002-2029-6844

### Data availability

All of the data that support the findings of this study are available in the main text or Supplementary Information.

## Supplementary materials

Supplementary material 1 Alignment file Data type fas https://binary.pensoft.net/file/1690112This dataset is made available under the Open Database License (http://opendatacommons.org/licenses/odbl/1.0/). The Open Database License (ODbL) is a license agreement intended to allow users to freely share, modify, and use this Dataset while maintaining this same freedom for others, provided that the original source and author(s) are credited. Xiu-Hong Xu, Xing Mo, A-Min Chen, Ya-Dong Li, Yun Pei, Jing-Wei Li, Ting-Chi Wen, Samantha C. Karunarathna, Entaj Tarafder, Wan-Ping Zhang

Supplementary material 2 AB1 file Data type zip https://binary.pensoft.net/file/1690113This dataset is made available under the Open Database License (http://opendatacommons.org/licenses/odbl/1.0/). The Open Database License (ODbL) is a license agreement intended to allow users to freely share, modify, and use this Dataset while maintaining this same freedom for others, provided that the original source and author(s) are credited. Xiu-Hong Xu, Xing Mo, A-Min Chen, Ya-Dong Li, Yun Pei, Jing-Wei Li, Ting-Chi Wen, Samantha C. Karunarathna, Entaj Tarafder, Wan-Ping Zhang
