# Supplementary material for: Morphological and molecular evidence reveal two new species of Sutorius (Boletaceae, Basidiomycota) from Guizhou Province, China
Source: MycoKeys. 2026 Jun 22;134:275–90. doi: 10.3897/mycokeys.134.192749 (PMC13316140; doi:10.3897/mycokeys.134.192749)
Supplement: Supplementary material 2 — AB1 file [file mycokeys-134-275-s002.zip › AB1/Sutorius rhodocapus/0040_31525092200153_(Xu117-bRPB2)_[bRPB2-7.1R]_H.pdf]

File: 0040_31525092200153_(Xu117-bRPB2)_[bRPB2-7.1R].ab1 Run Ended: 2025-09-26 06:09:17 Signal G:459 A:982 T:1152 C:1566

Sample: 71505860973_31525092200153_(Xu117-bRPB2)_[bRPB2-7.1R]_JZ Lane: 4 Base spacing: 15.1 776 bases in 9560 scans

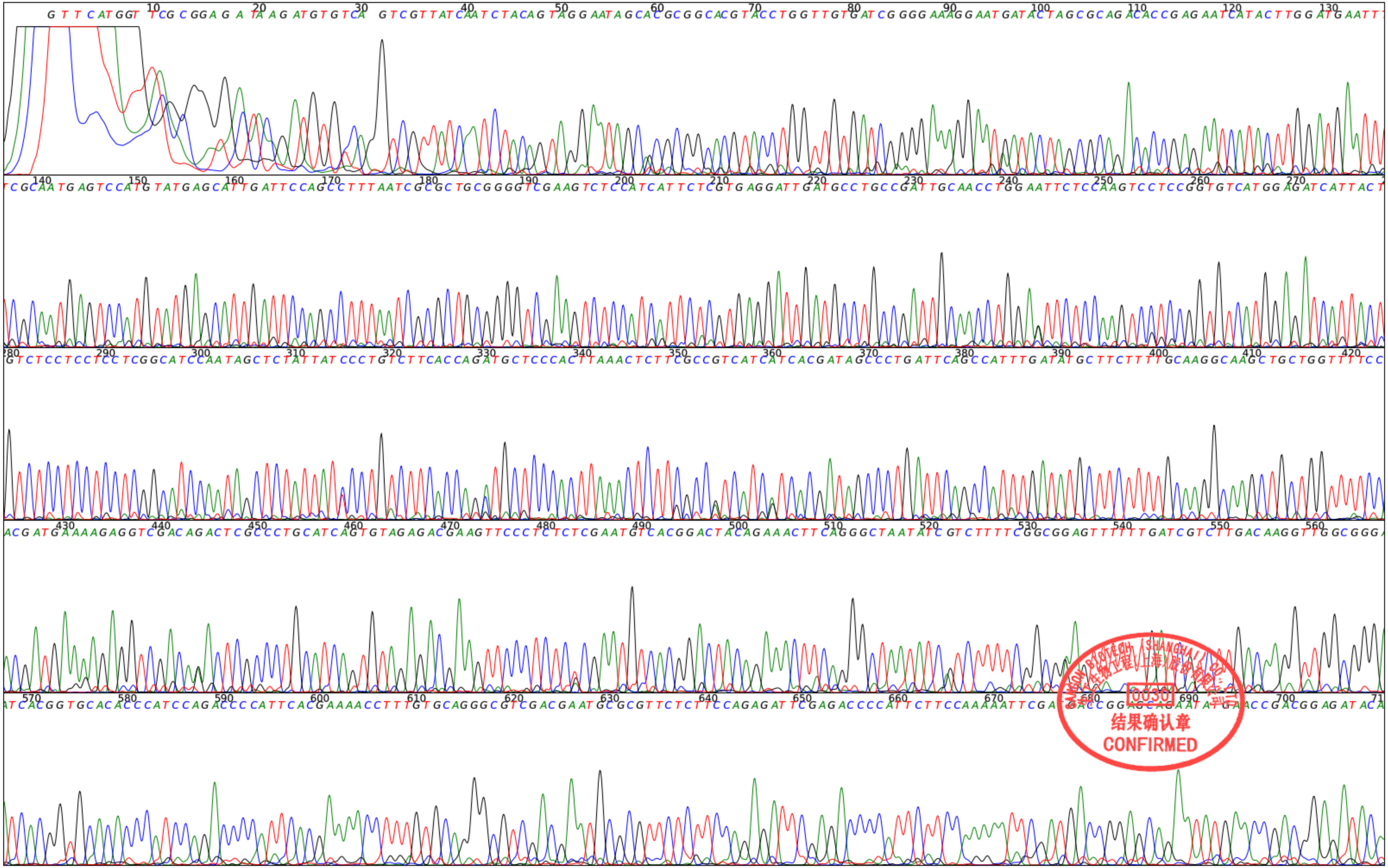

File: 0040_31525092200153_(Xu117-bRPB2)_[bRPB2-7.1R].ab1 Run Ended: 2025-09-26 06:09:17 Signal G:459 A:982 T:1152 C:1566

Sample: 71505860973_31525092200153_(Xu117-bRPB2)_[bRPB2-7.1R]_JZ Lane: 4 Base spacing: 15.1 776 bases in 9560 scans

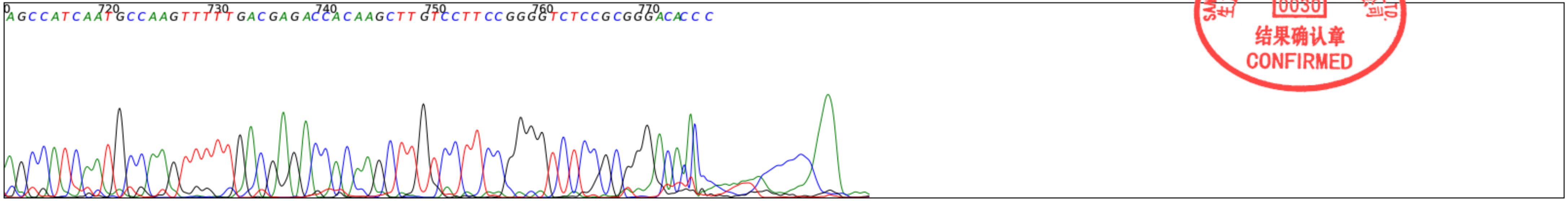

SANGON BIOTECH (SHANGHAI) CO., LTD.
生工生物工程(上海)股份有限公司
0030
结果确认章
CONFIRMED
