# Supplementary figures and images for: Morphological and molecular evidence reveal two new species of Sutorius (Boletaceae, Basidiomycota) from Guizhou Province, China
Source: MycoKeys. 2026 Jun 22;134:275–90. doi: 10.3897/mycokeys.134.192749 (PMC13316140; doi:10.3897/mycokeys.134.192749)

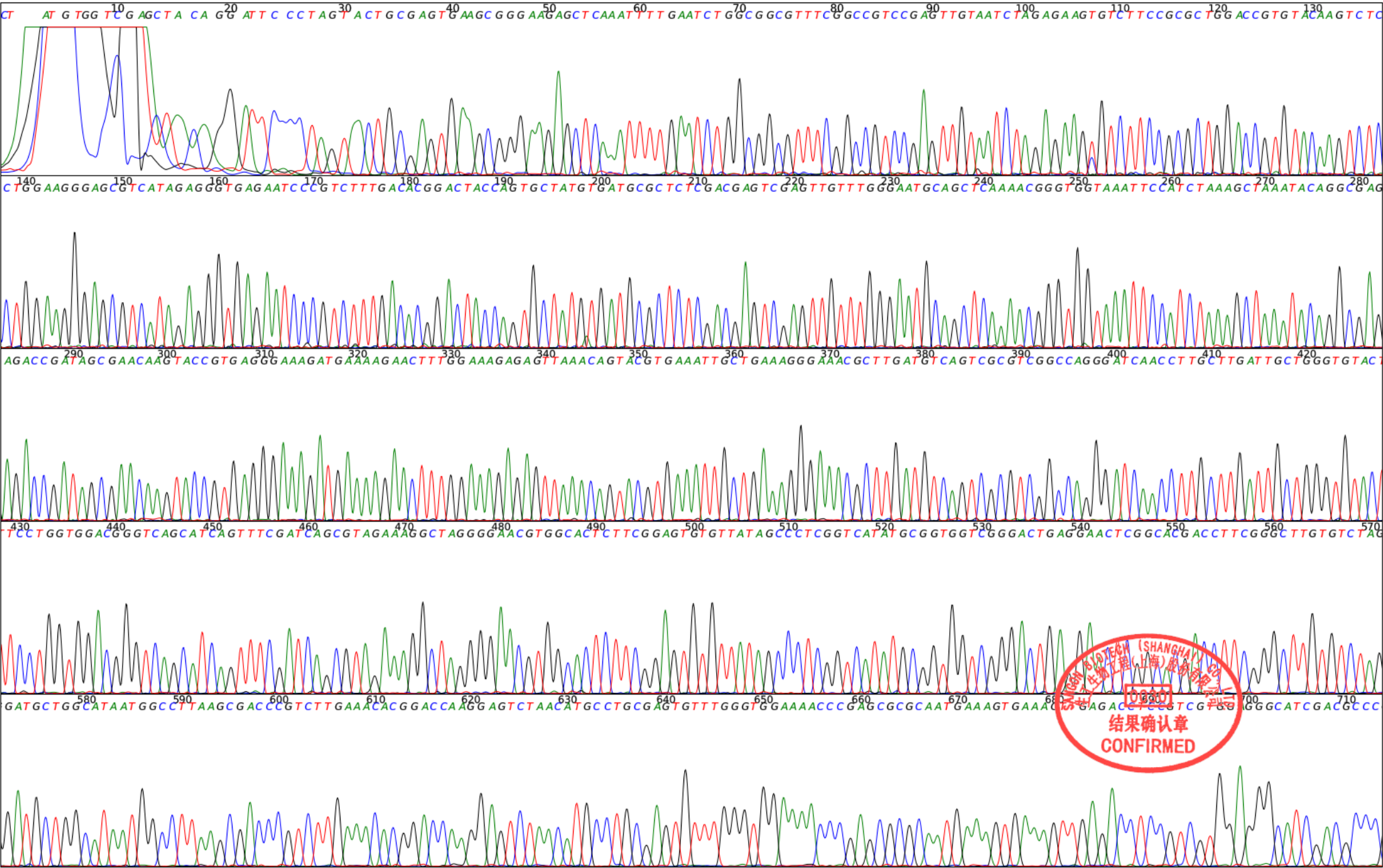

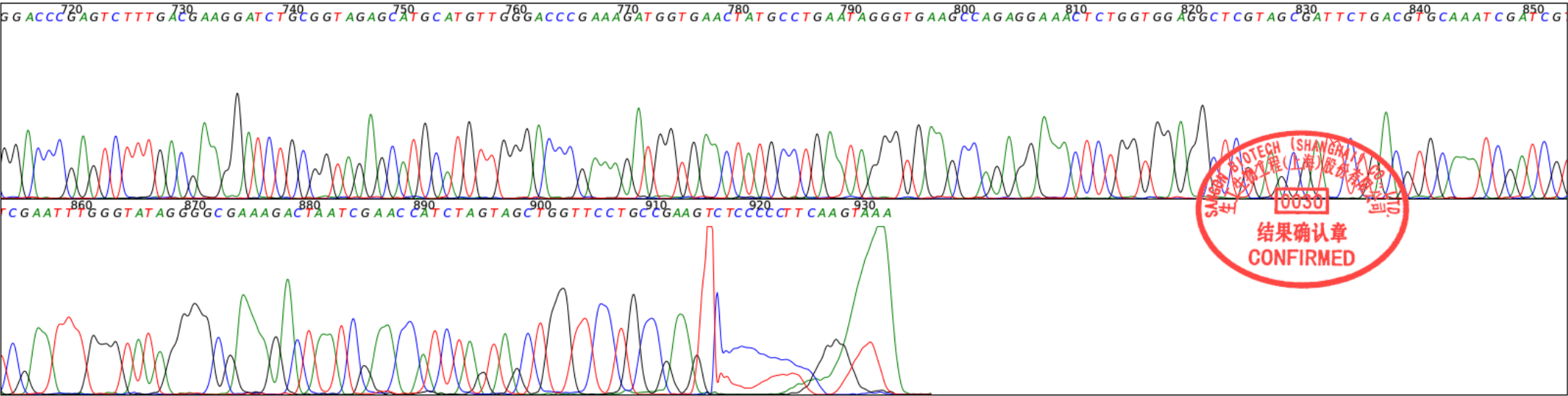

Supplement: Supplementary material 2 — AB1 file [file mycokeys-134-275-s002.zip › AB1/Sutorius rhodocapus/0003_31525092200135_(Xu117-LSU)_[LR0RF]_H.pdf]

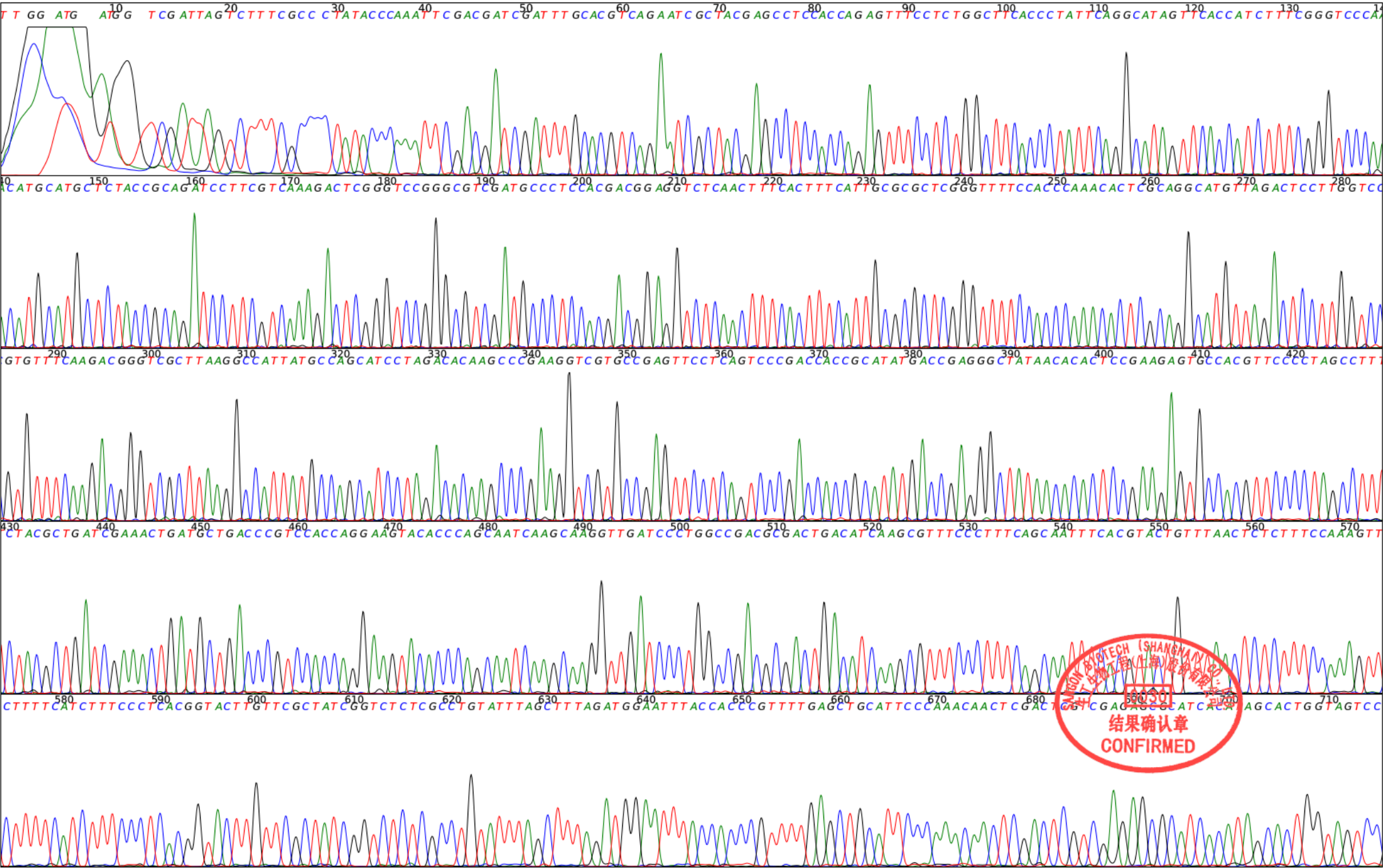

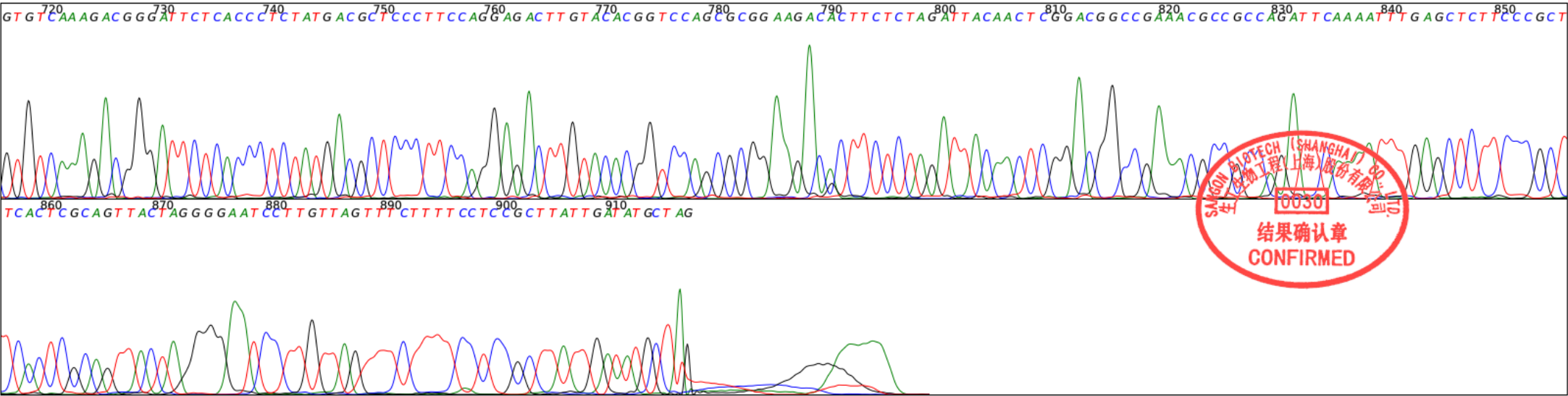

Supplement: Supplementary material 2 — AB1 file [file mycokeys-134-275-s002.zip › AB1/Sutorius rhodocapus/0004_31525092200135_(Xu117-LSU)_[LR5R]_H.pdf]

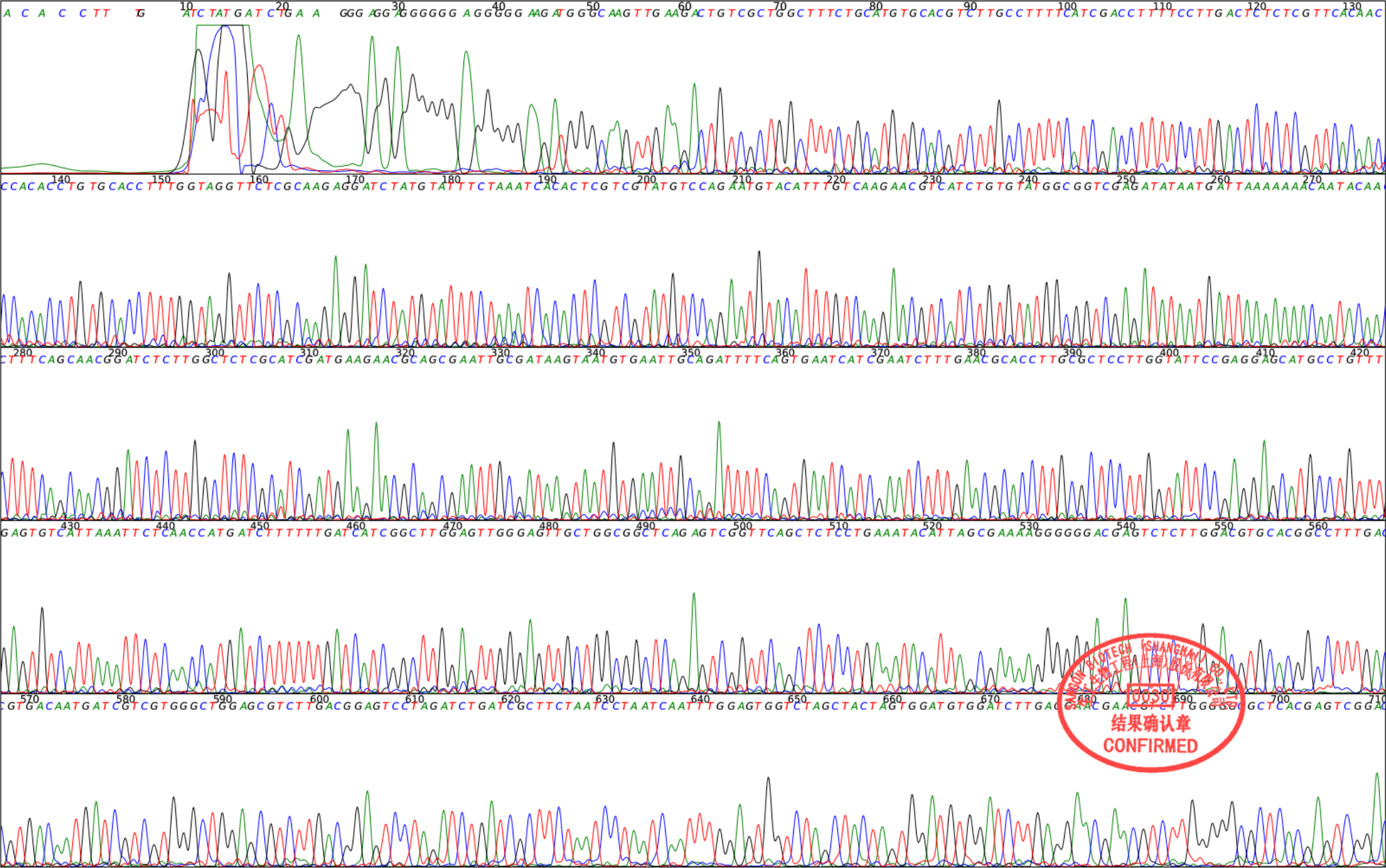

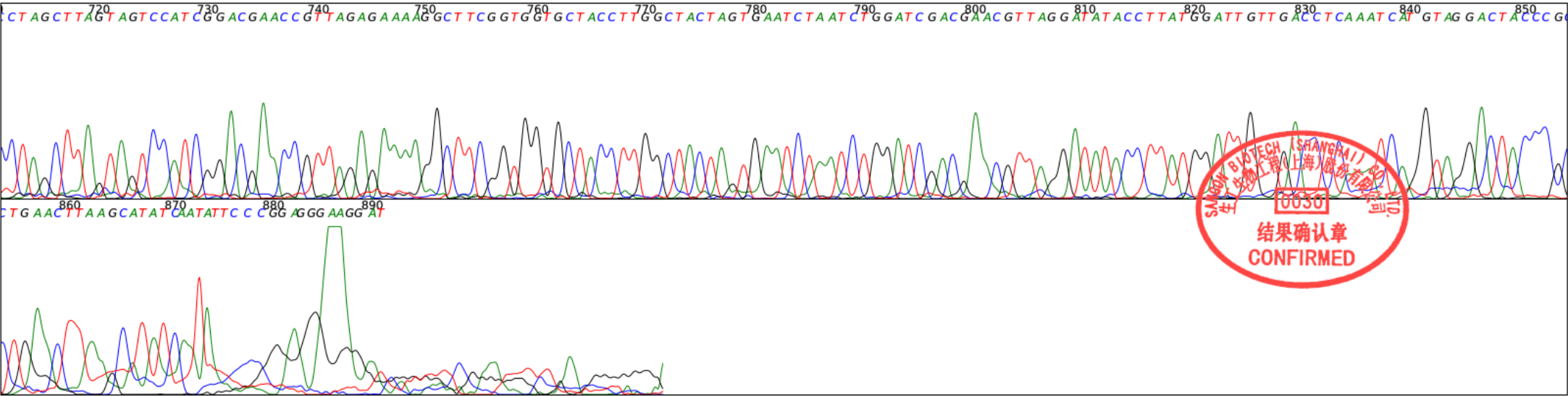

Supplement: Supplementary material 2 — AB1 file [file mycokeys-134-275-s002.zip › AB1/Sutorius rhodocapus/0015_31525092200141_(Xu117-ITS)_[ITS1]_H.pdf]

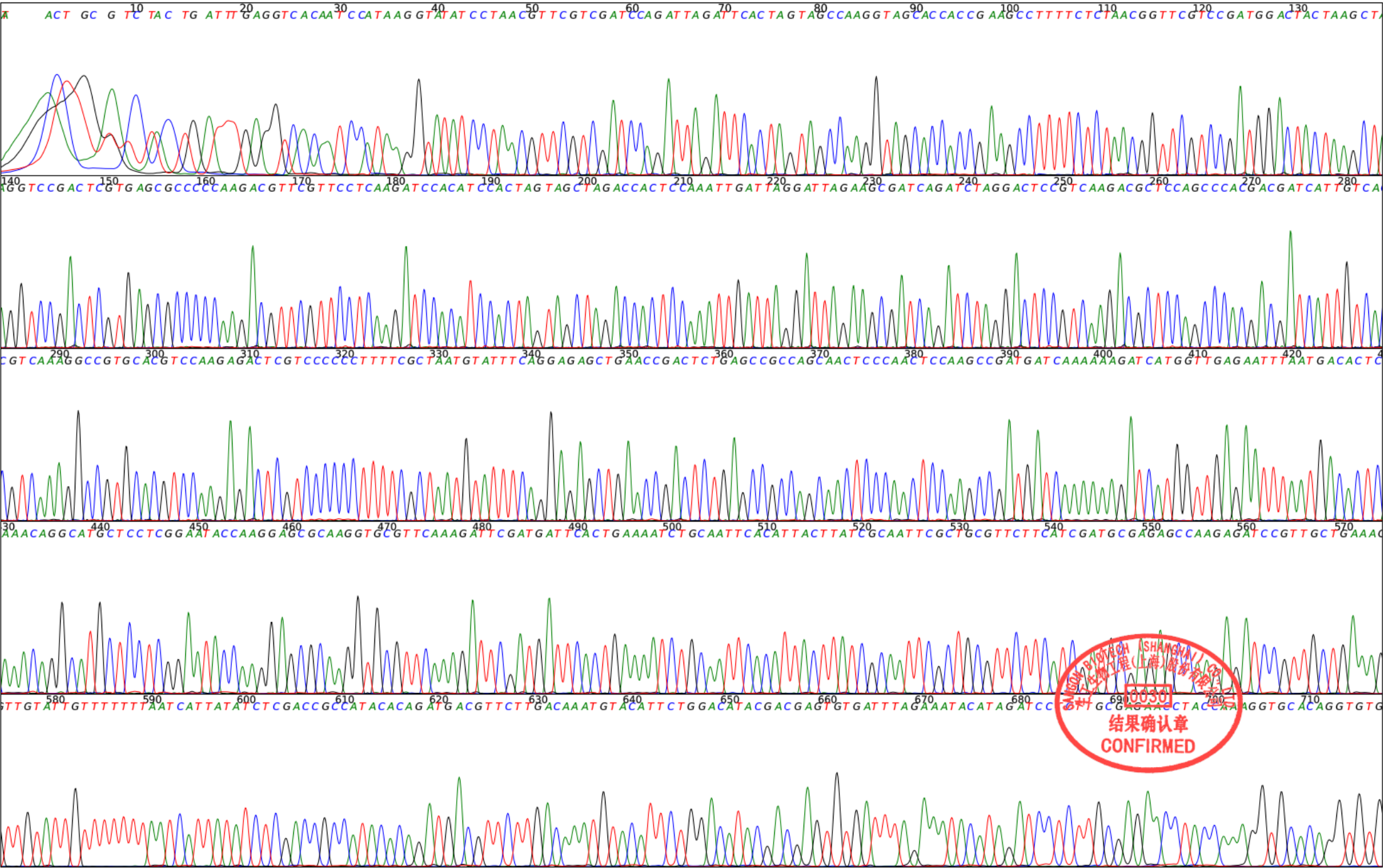

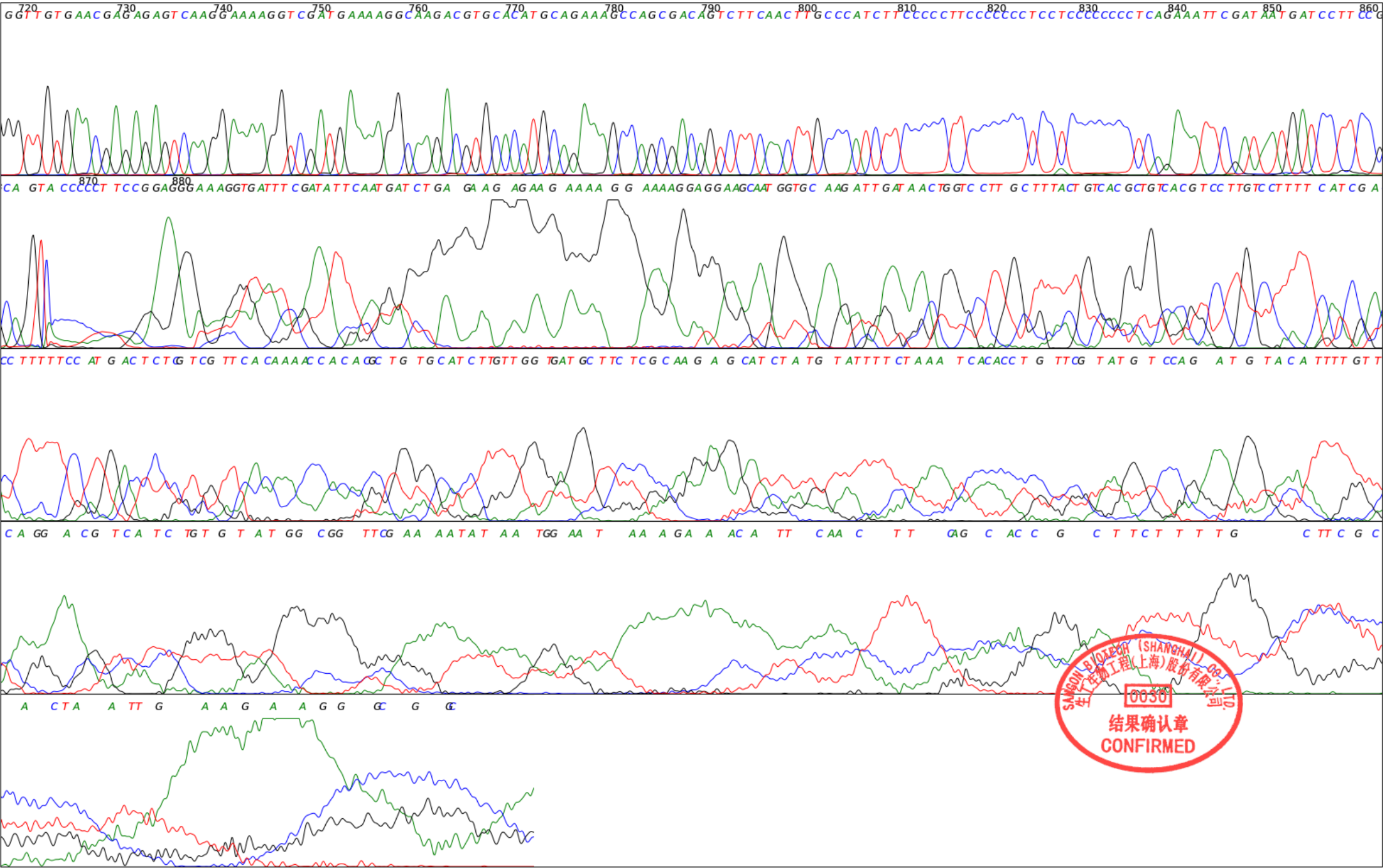

Supplement: Supplementary material 2 — AB1 file [file mycokeys-134-275-s002.zip › AB1/Sutorius rhodocapus/0016_31525092200141_(Xu117-ITS)_[ITS4]_H.pdf]

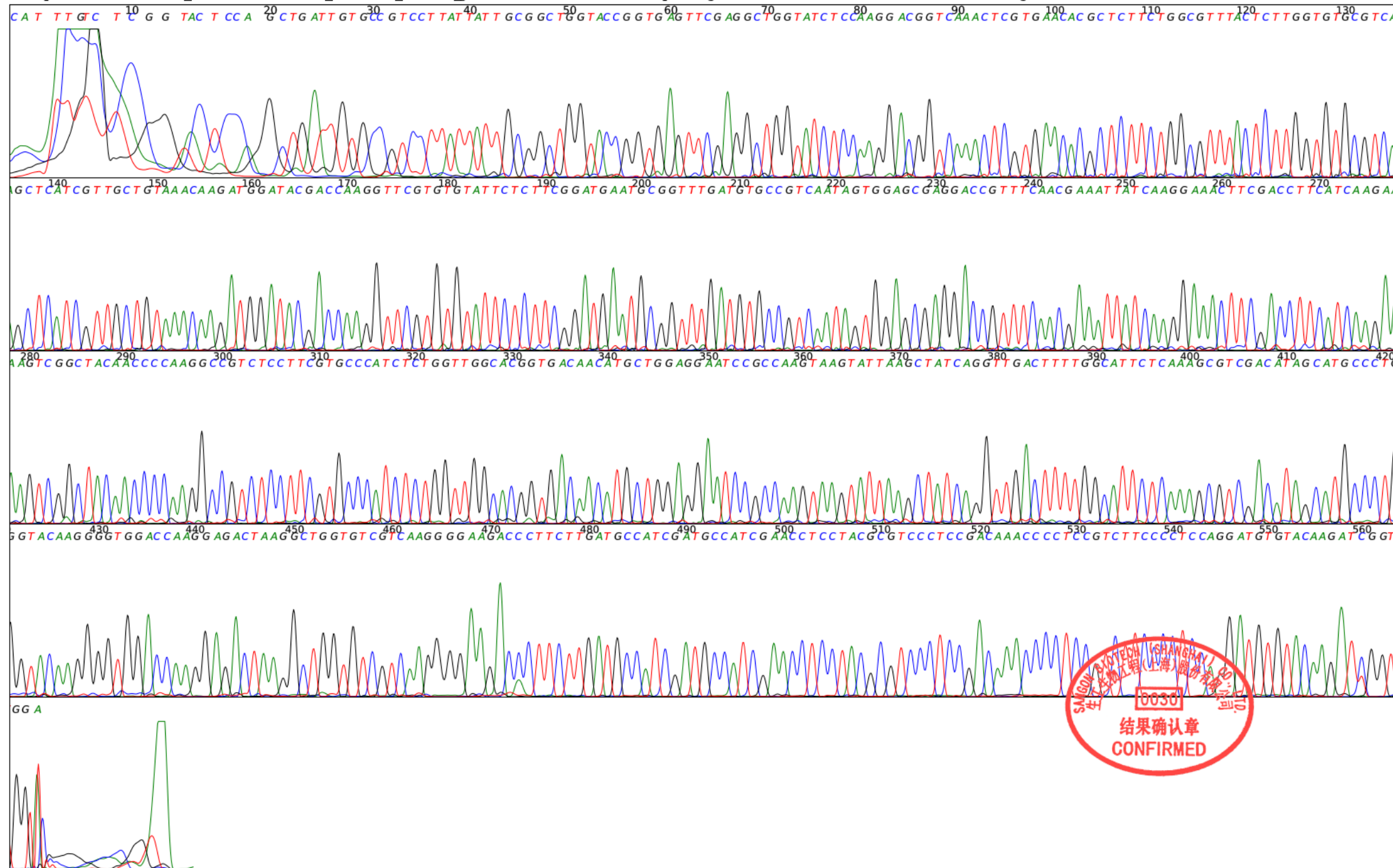

Supplement: Supplementary material 2 — AB1 file [file mycokeys-134-275-s002.zip › AB1/Sutorius rhodocapus/0027_31525092200147_(Xu117)_[983F]_H.pdf]

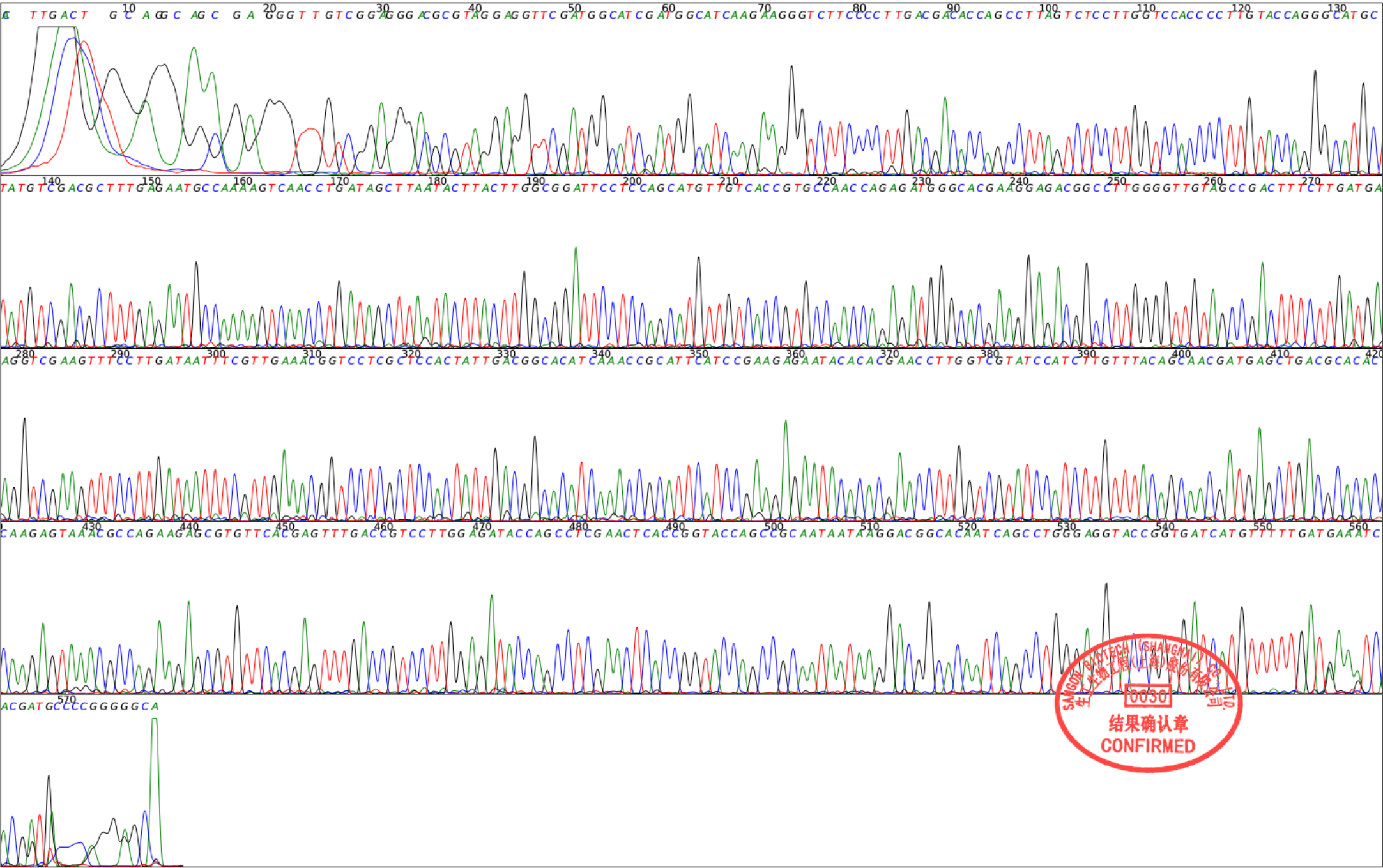

Supplement: Supplementary material 2 — AB1 file [file mycokeys-134-275-s002.zip › AB1/Sutorius rhodocapus/0028_31525092200147_(Xu117)_[1567R]_H.pdf]

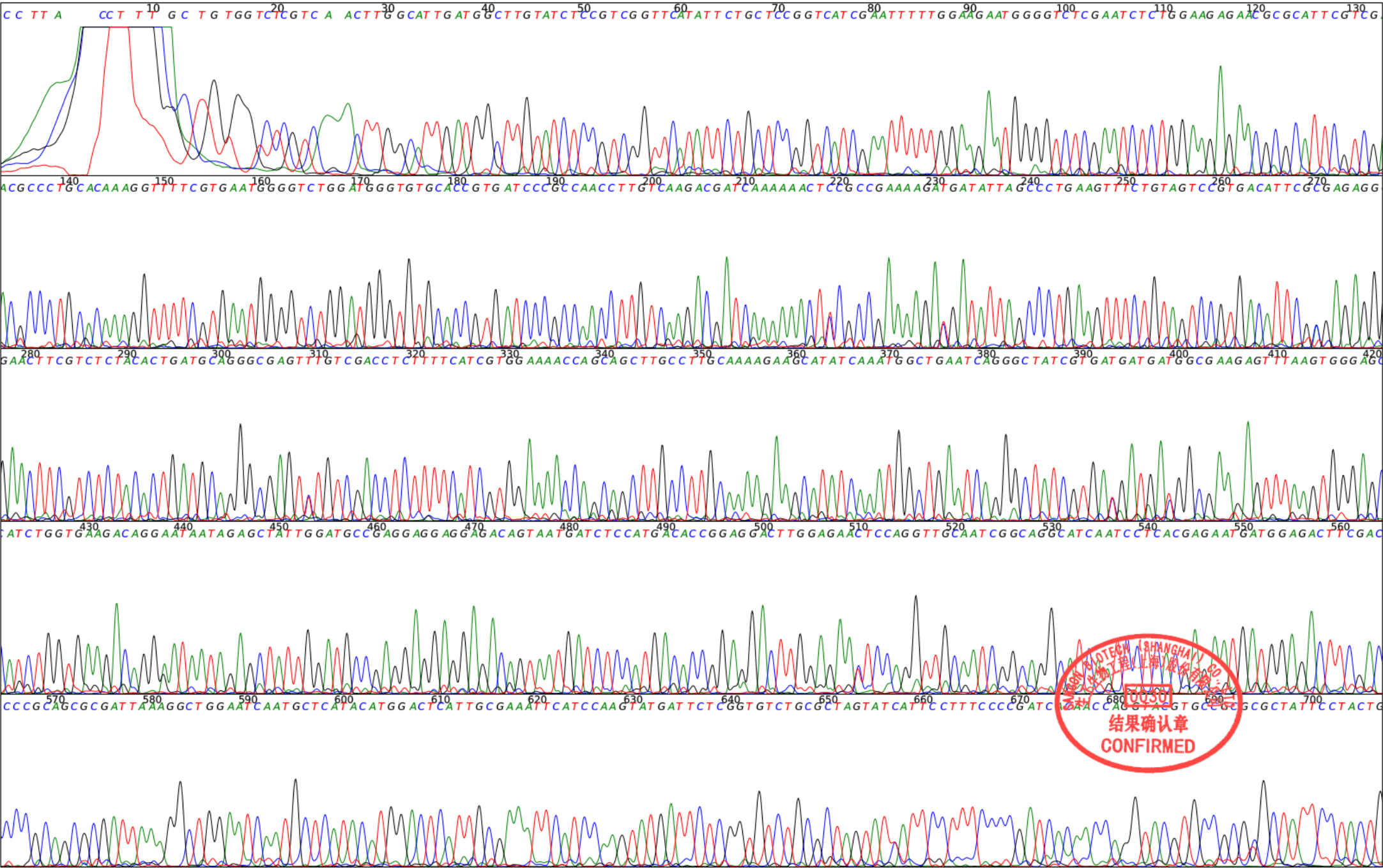

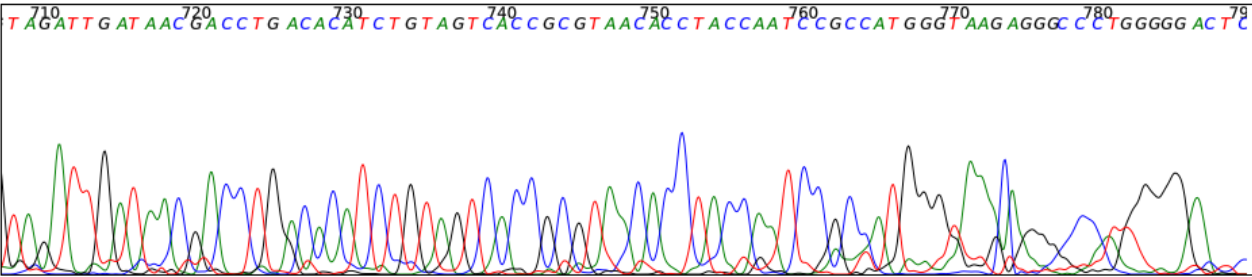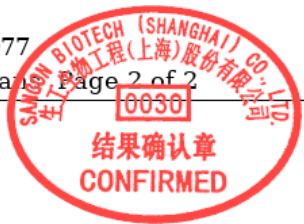

Supplement: Supplementary material 2 — AB1 file [file mycokeys-134-275-s002.zip › AB1/Sutorius rhodocapus/0039_31525092200153_(Xu117-bRPB2)_[bRPB2-6F]_H.pdf]

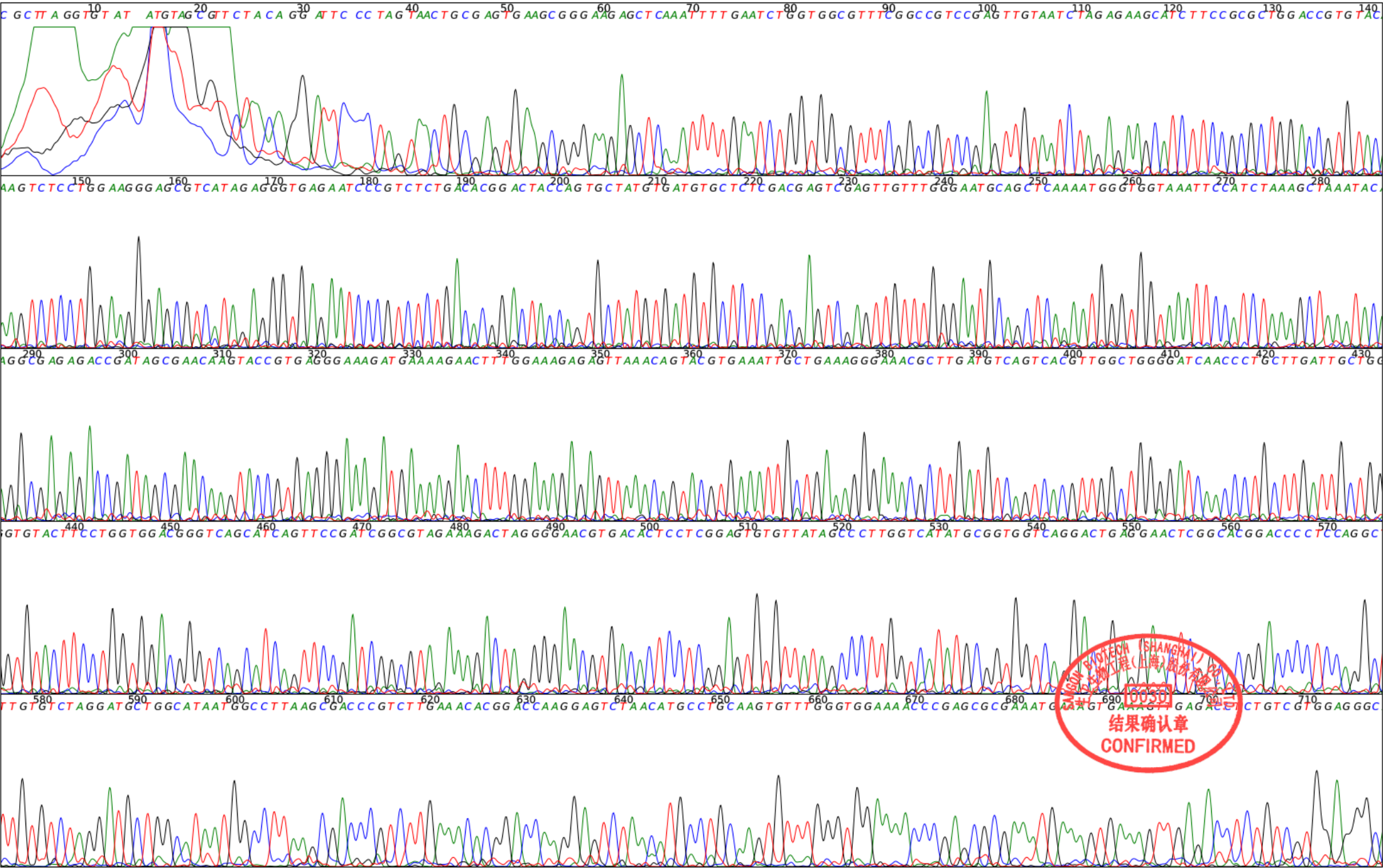

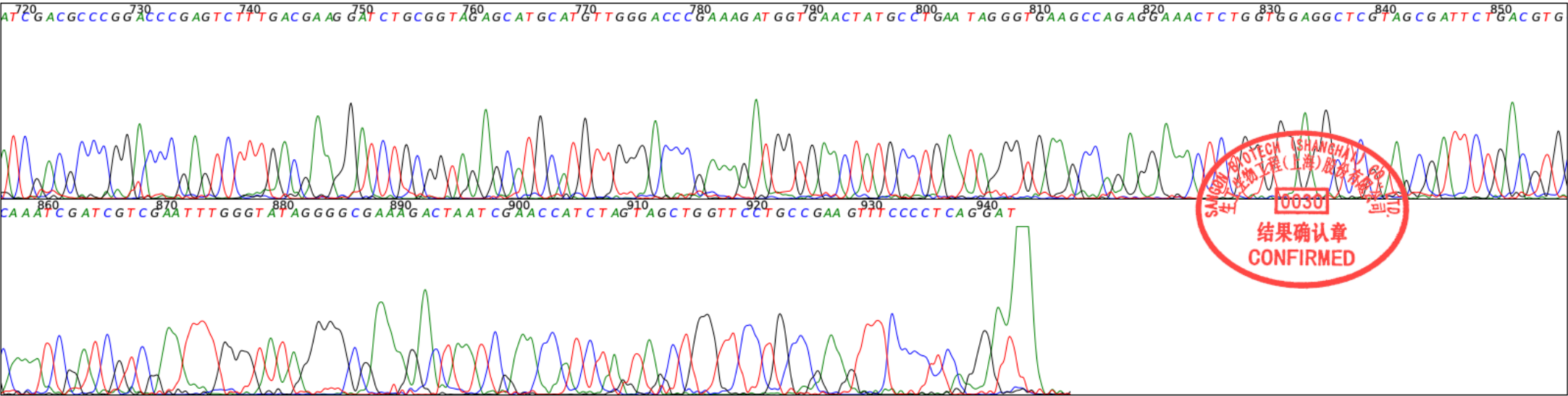

Supplement: Supplementary material 2 — AB1 file [file mycokeys-134-275-s002.zip › AB1/Sutorius yuxiensis/0011_31525092200139_(Xu186-LSU)_[LR0RF]_H.pdf]

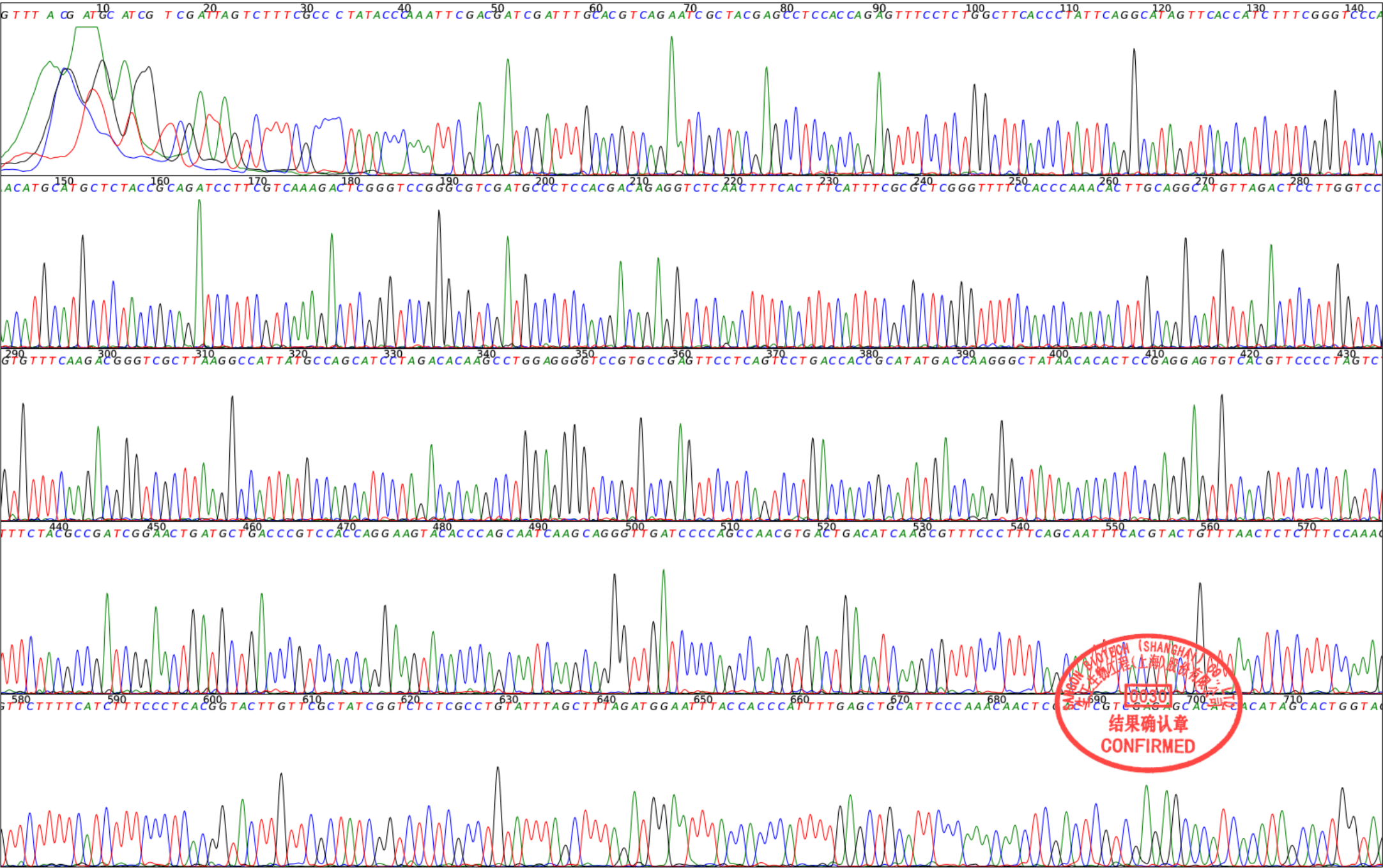

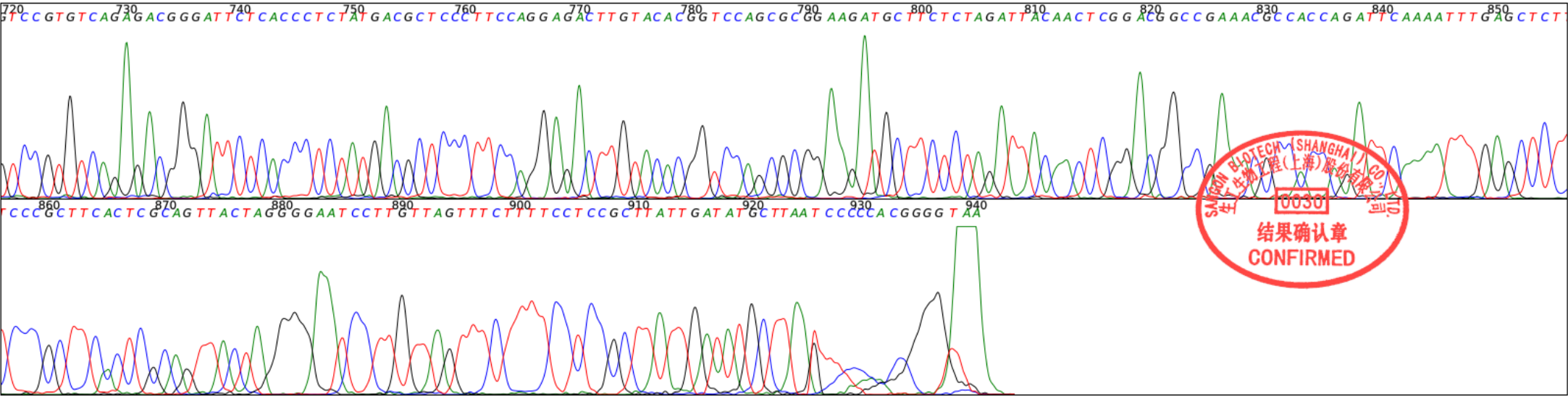

Supplement: Supplementary material 2 — AB1 file [file mycokeys-134-275-s002.zip › AB1/Sutorius yuxiensis/0012_31525092200139_(Xu186-LSU)_[LR5R]_H.pdf]

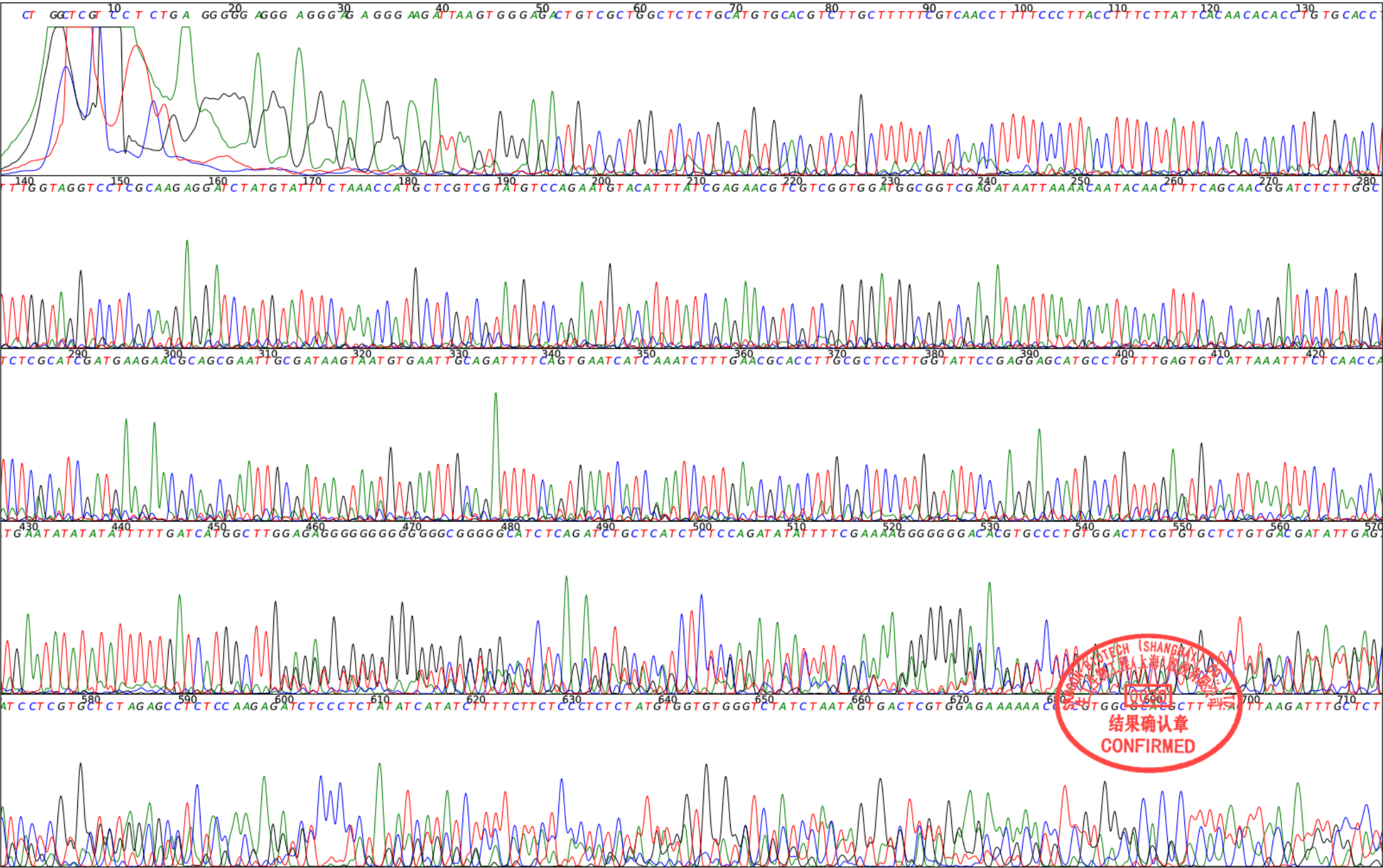

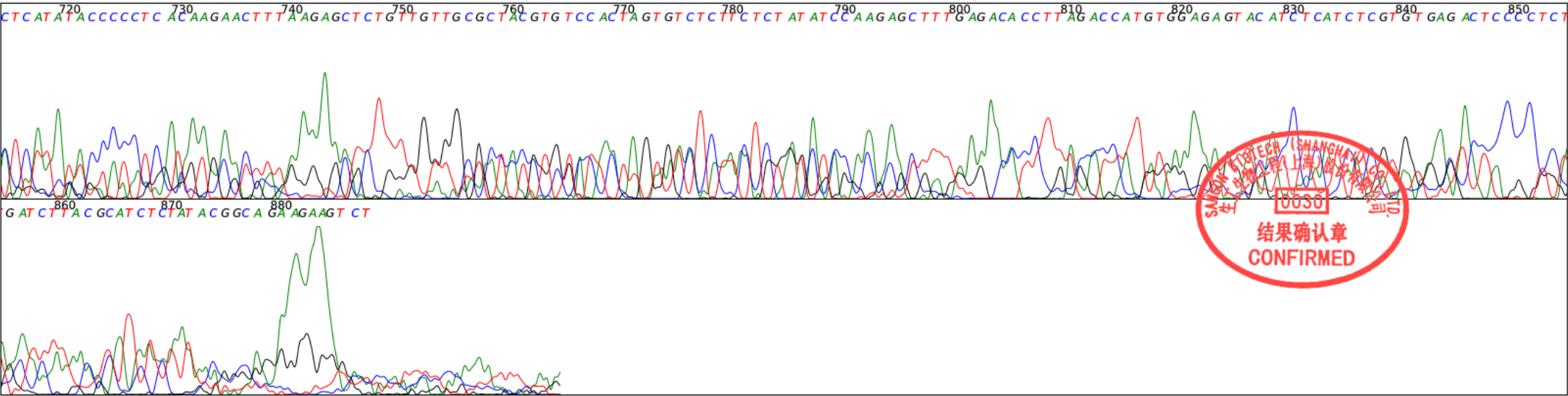

Supplement: Supplementary material 2 — AB1 file [file mycokeys-134-275-s002.zip › AB1/Sutorius yuxiensis/0023_31525092200145_(Xu186-ITS)_[ITS1]_H.pdf]

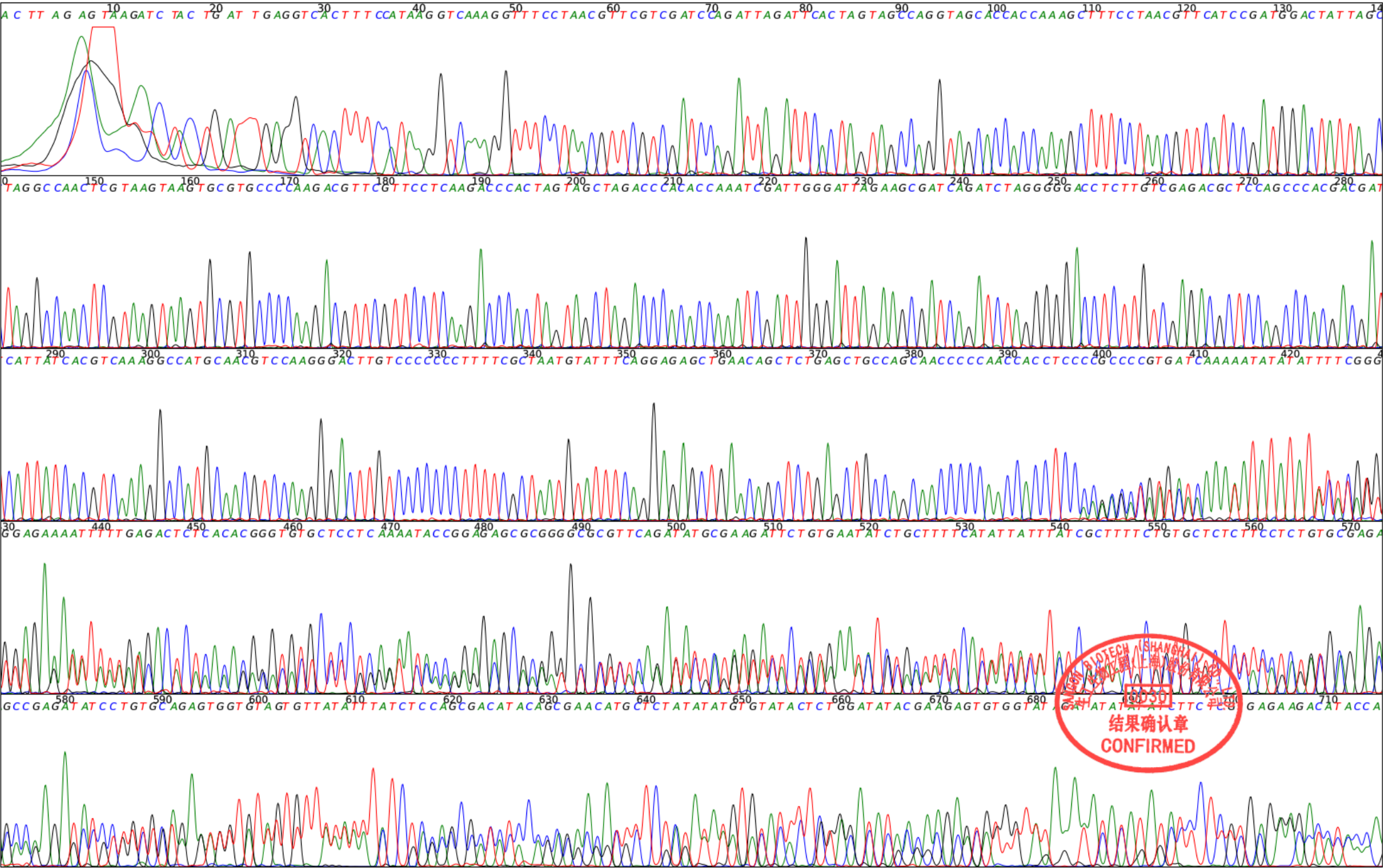

BIOTECH (SHANGHAI)  
结果确认章  
CONFIRMED

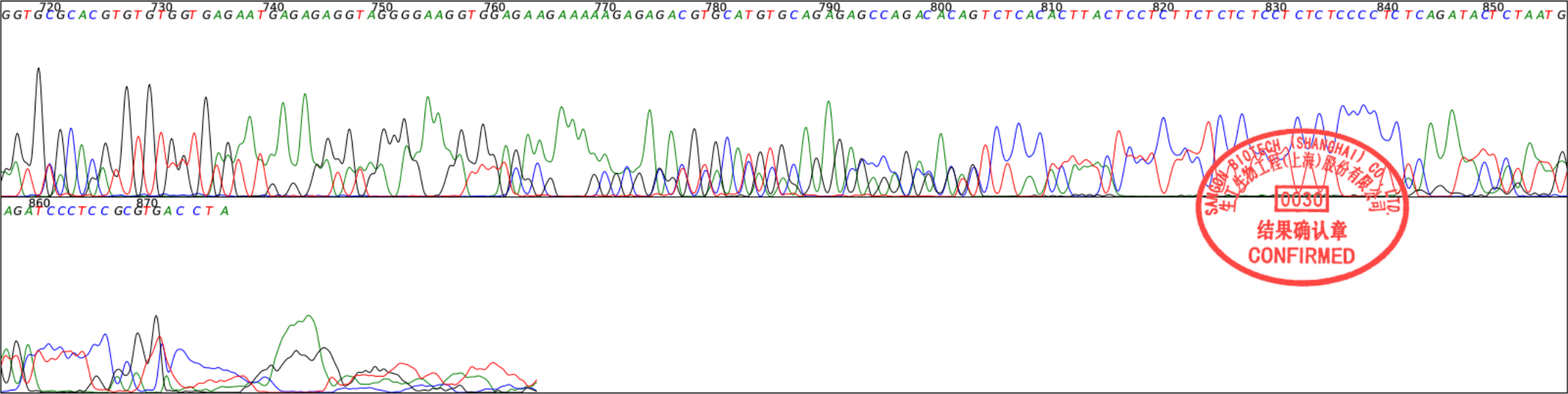

Supplement: Supplementary material 2 — AB1 file [file mycokeys-134-275-s002.zip › AB1/Sutorius yuxiensis/0024_31525092200145_(Xu186-ITS)_[ITS4]_H.pdf]

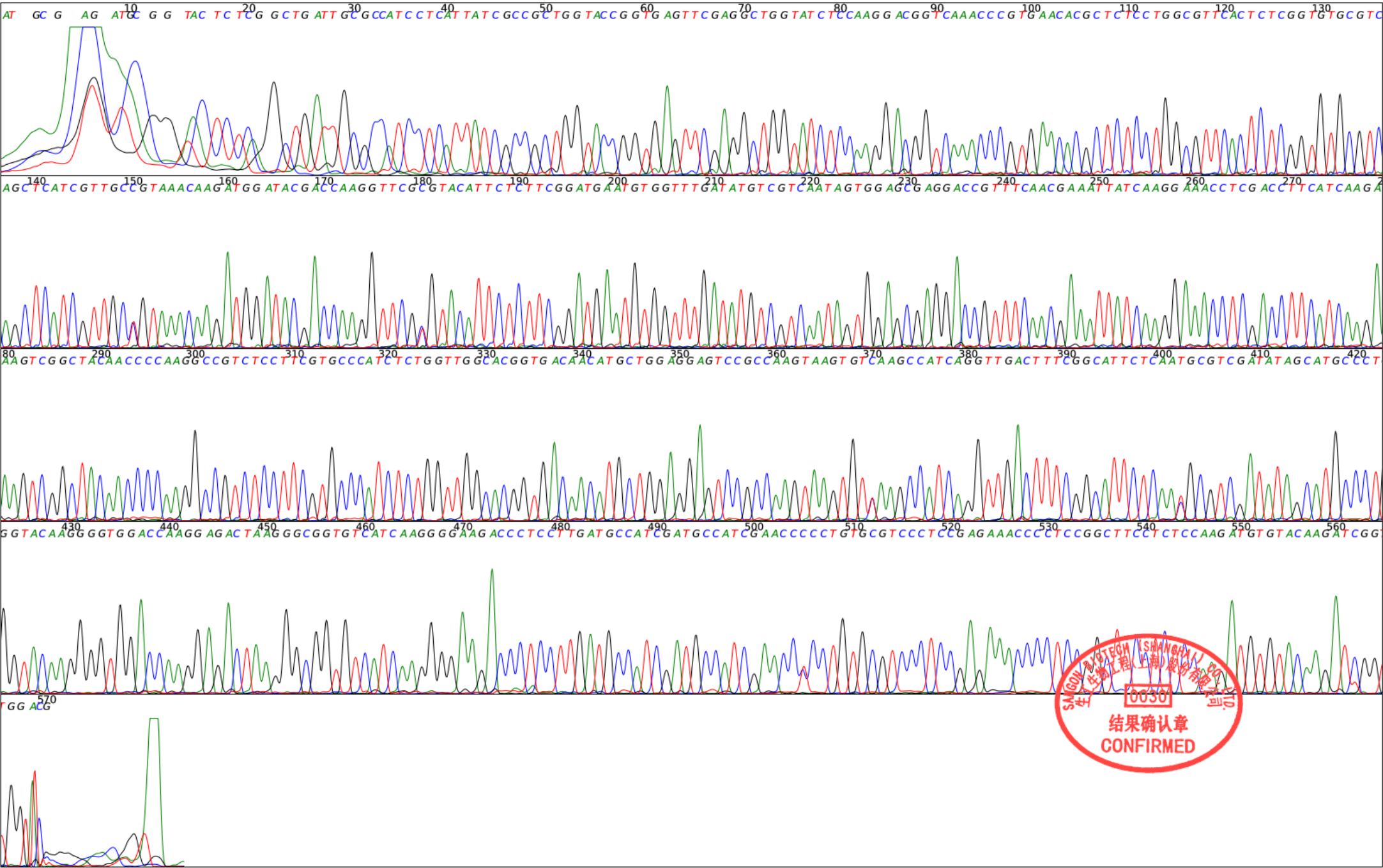

SAISON VETECH (SHANGHAI) CO., LTD.  
0035  
结果确认章  
CONFIRMED

Supplement: Supplementary material 2 — AB1 file [file mycokeys-134-275-s002.zip › AB1/Sutorius yuxiensis/0035_31525092200151_(Xu186)_[983F]_H.pdf]

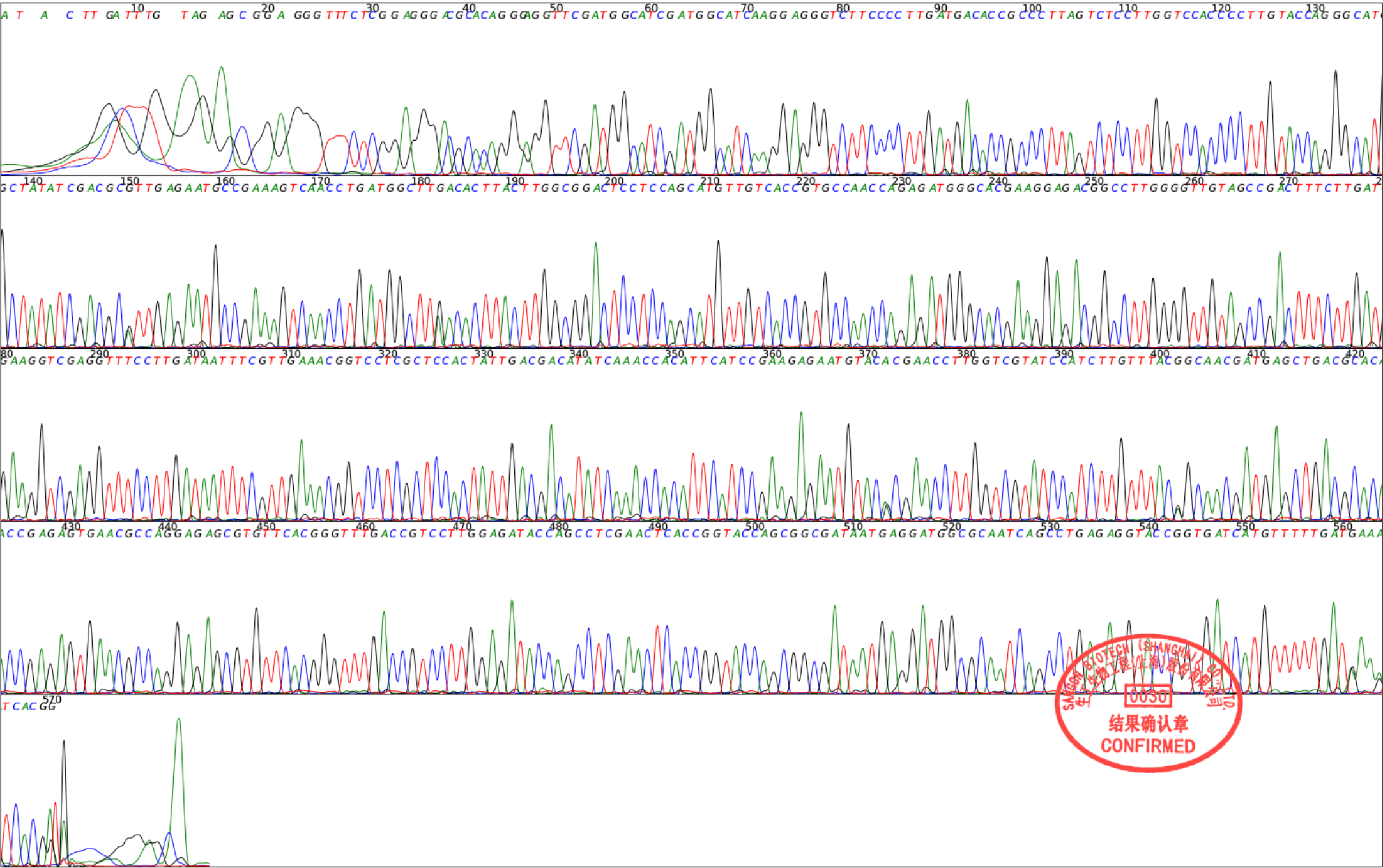

Supplement: Supplementary material 2 — AB1 file [file mycokeys-134-275-s002.zip › AB1/Sutorius yuxiensis/0036_31525092200151_(Xu186)_[1567R]_H.pdf]

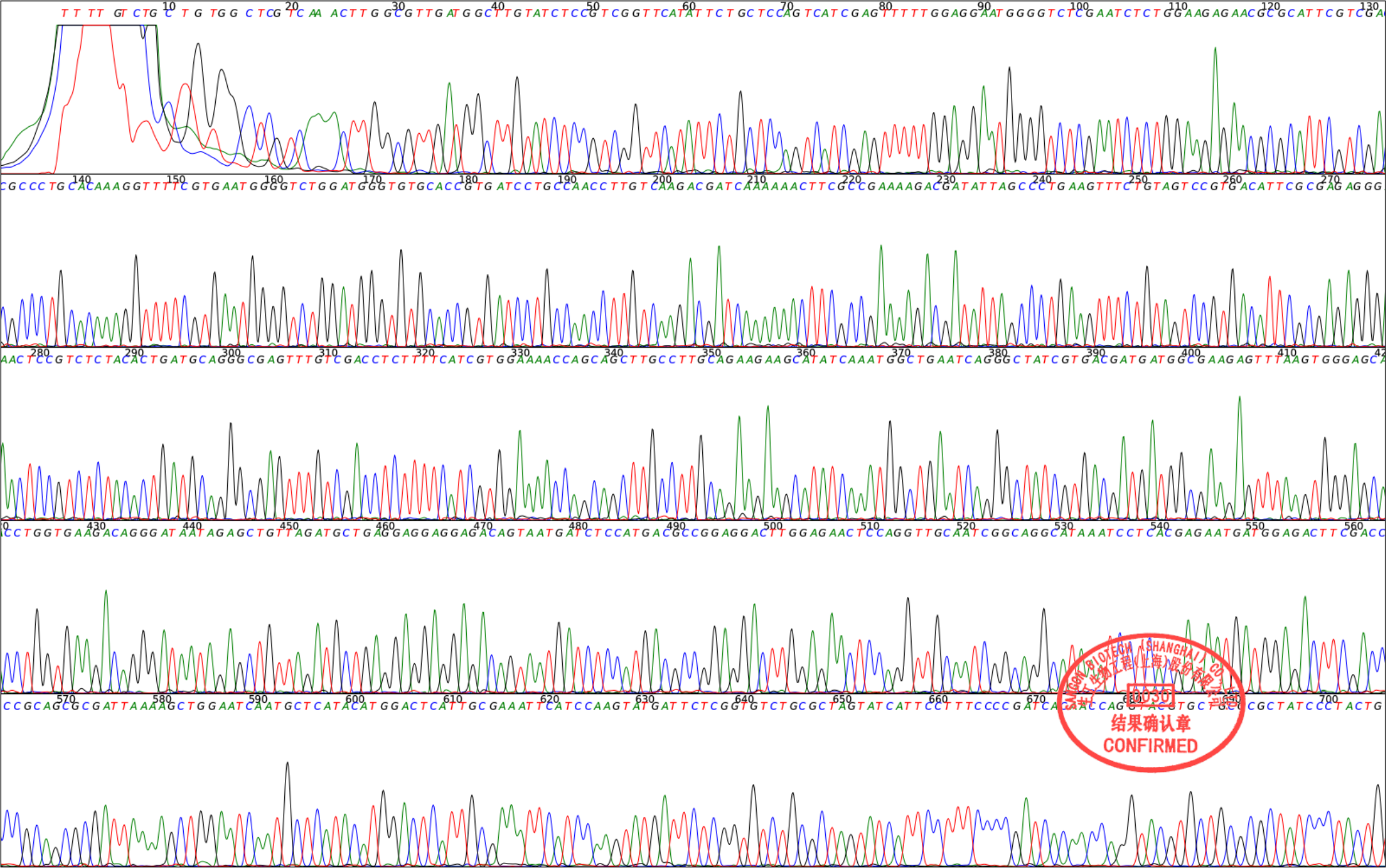

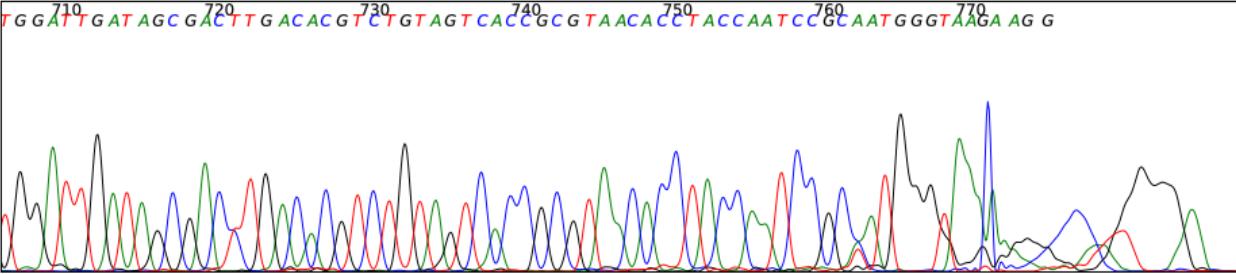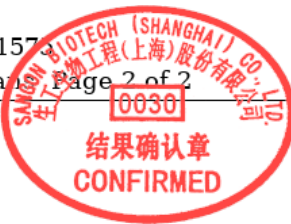

Supplement: Supplementary material 2 — AB1 file [file mycokeys-134-275-s002.zip › AB1/Sutorius yuxiensis/0047_31525092200157_(Xu186-bRPB2)_[bRPB2-6F]_H.pdf]

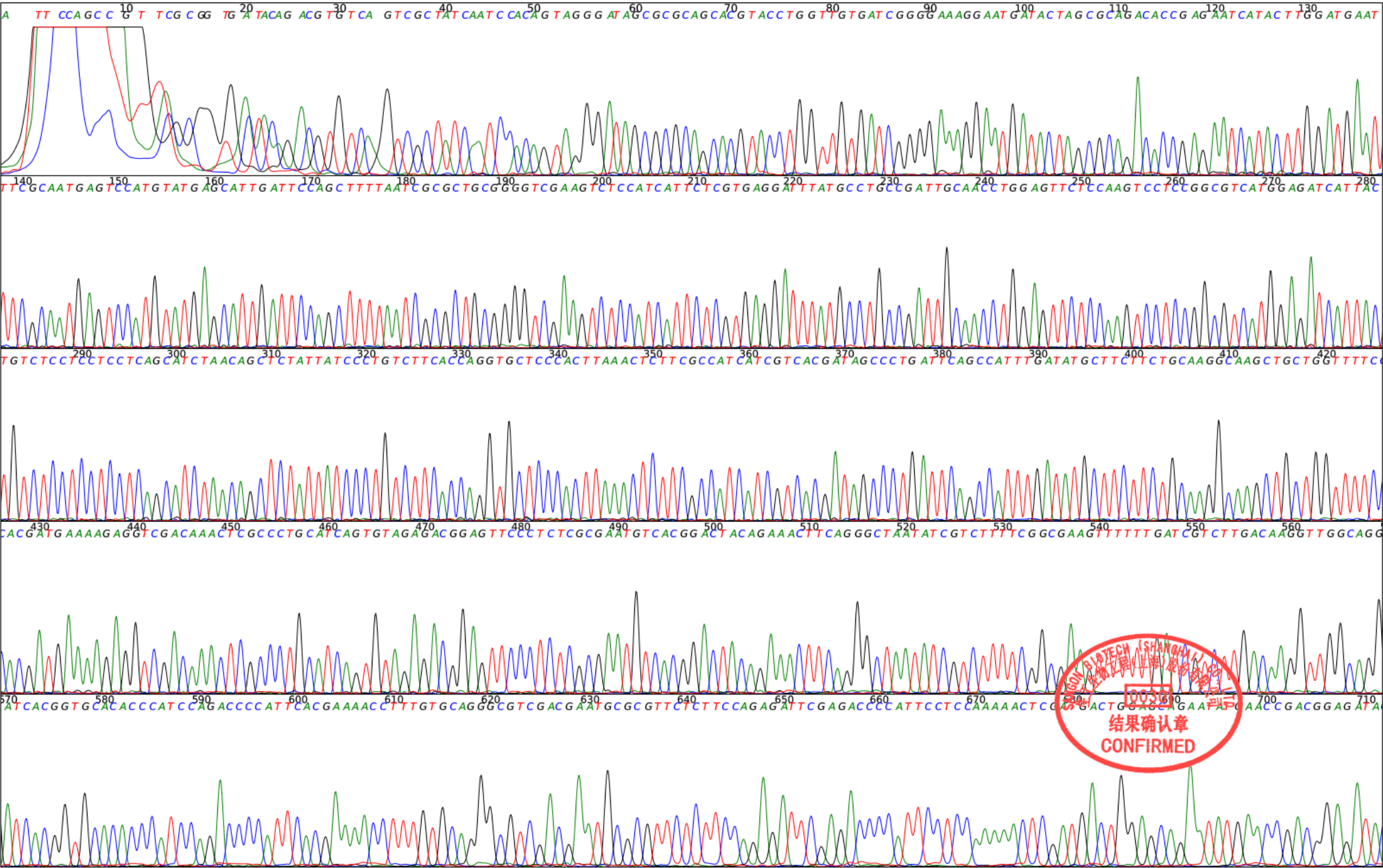

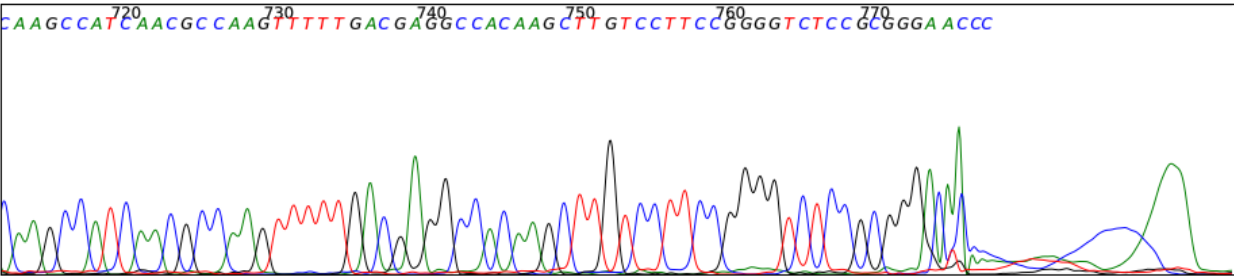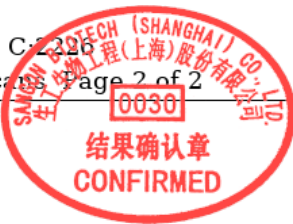

Supplement: Supplementary material 2 — AB1 file [file mycokeys-134-275-s002.zip › AB1/Sutorius yuxiensis/0048_31525092200157_(Xu186-bRPB2)_[bRPB2-7.1R]_H.pdf]
